# Supplementary figures and images for: Development and characterization of microsatellite markers for population genetics of the cocoa pod borer Conopomorpha cramerella (Snellen) (Lepidoptera: Gracillaridae)
Source: PLoS One. 2024 Apr 11;19(4):e0297662. doi: 10.1371/journal.pone.0297662 (PMC11008836; doi:10.1371/journal.pone.0297662)

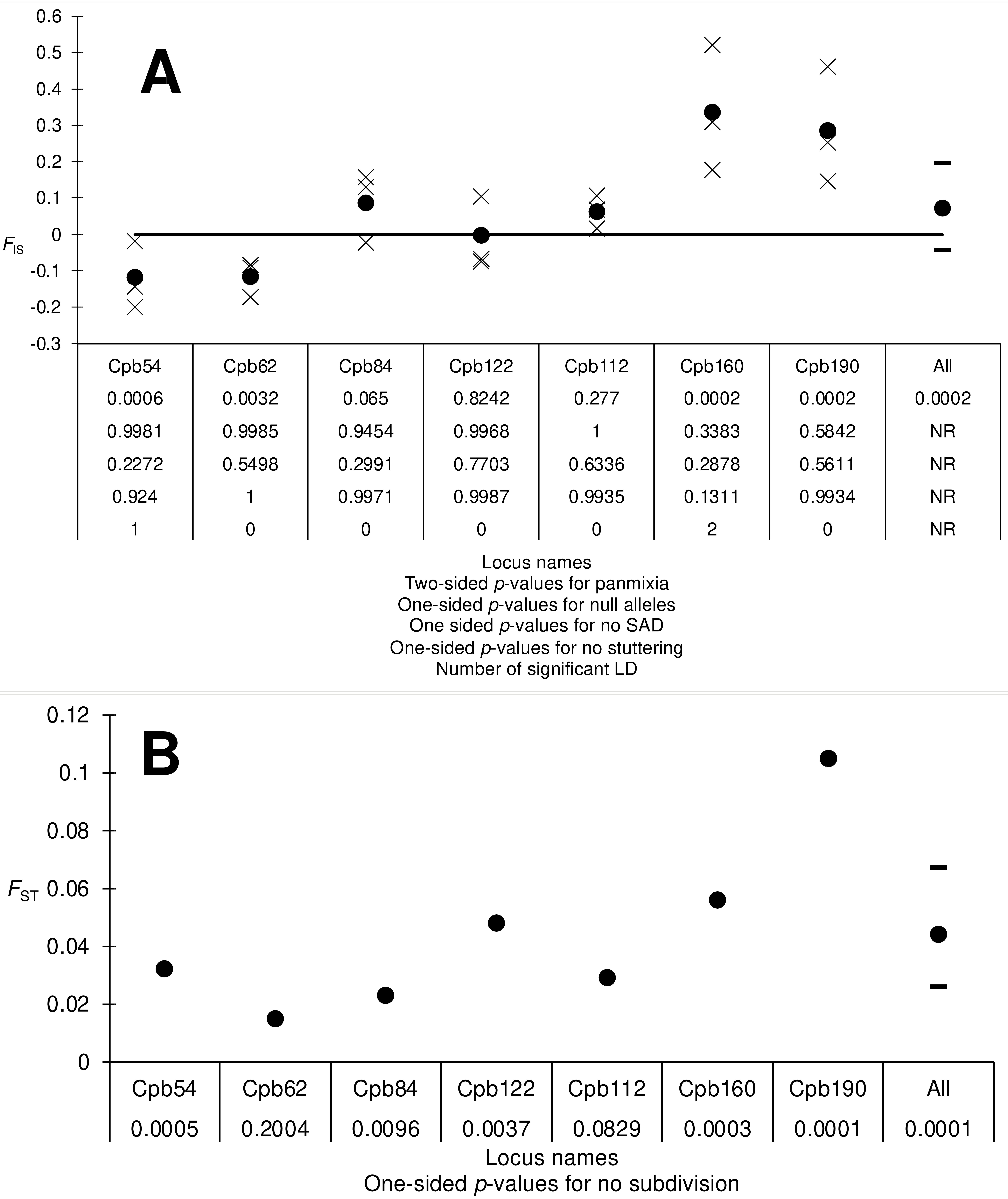

Supplement: S1 Fig — (A) Wright’s FIS with p-values for panmixia, null alleles, SAD, stuttering and null alleles and (B) FST with p-values for subdivision of the seven selected microsatellite loci of cocoa pod borer Conopomorpha cramerella. [•]: FIS or FST per locus, [x]: FIS per population [-]: 95% CI. (TIF) [file pone.0297662.s007.tif]

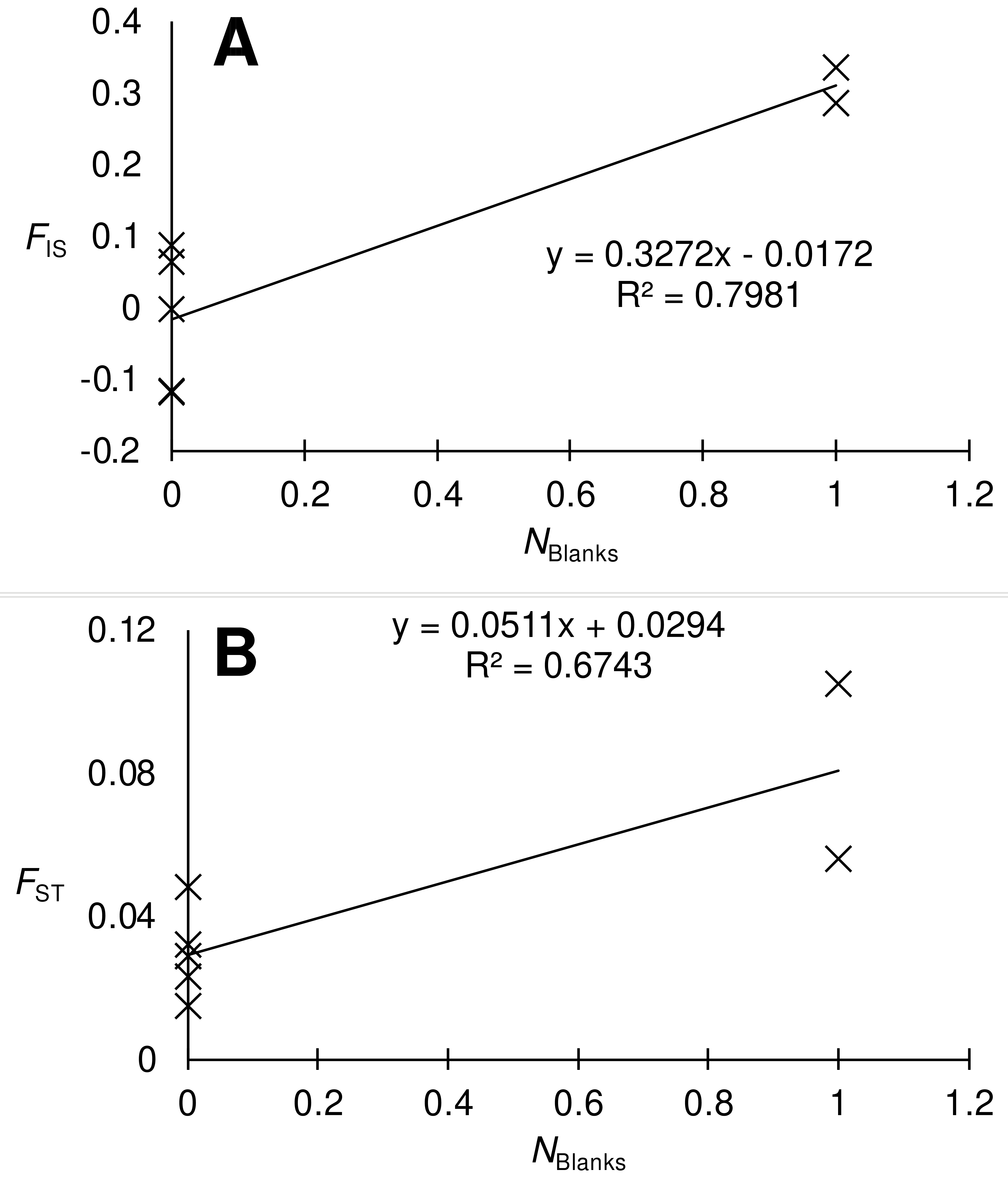

Supplement: S2 Fig — The correlation shown is after assuming that the missing data for Cpb122 and Cpb62 are not null homozygotes. (TIF) [file pone.0297662.s008.tif]

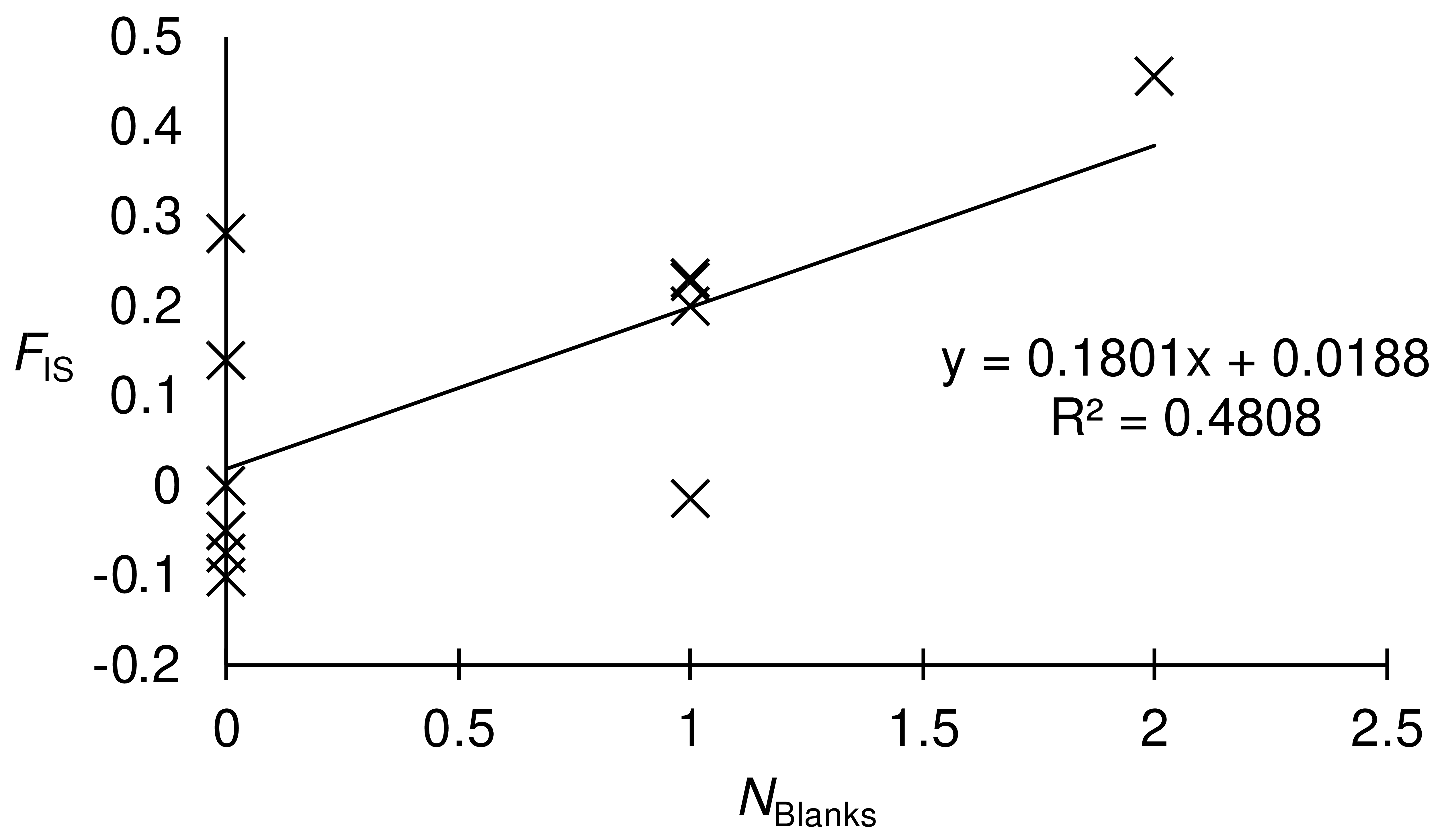

Supplement: S3 Fig — (TIF) [file pone.0297662.s009.tif]
