# Supplementary material for: Development and characterization of microsatellite markers for population genetics of the cocoa pod borer Conopomorpha cramerella (Snellen) (Lepidoptera: Gracillaridae)
Source: PLoS One. 2024 Apr 11;19(4):e0297662. doi: 10.1371/journal.pone.0297662 (PMC11008836; doi:10.1371/journal.pone.0297662)
Supplement: S1 File — (DOCX) [file pone.0297662.s010.docx]

**Development and characterization of microsatellite markers for population genetics of the cocoa pod borer *Conopomorpha cramerella* (Snellen) (Lepidoptera: Gracillaridae)**

Marynold Purificacion, Roslina Binti Mohd Shah, Thierry De Meeûs, Saripah Binti Bakar, Anisah Bintil Savantil, Meriam Mohd Yusof, Divina Amalin, Hien Nguyen, Endang Sulistyowati, Aris Budiman, Arni Ekayanti, Jerome Niogret, Sophie Ravel, Marc J.B. Vreysen, Adly M.M. Abd-Alla

2023-02-23

setwd("C:/Users/abdallaa/OneDrive - IAEA/My_passport_6/Marynold/analysis")

library(ggplot2)
library(MASS)
library(rmarkdown)
library(knitr)
library(lme4)
library(MuMIn)
library(ggthemes) # Load
library(datasets)
library(plyr)
library(dplyr)
library(tidyverse)
library(geosphere)

## Analysis CPB 11 loci; ajusted p value for LD

p.adjust(c(0.0125, 0.5208, 0.4659, 0.1141, 0.2034, 0.6403, 0.4994, 0.633, 0.8052, 0.4462, 0.0002, 0.8846, 0.5612, 0.0445, 0.2988, 0.9458, 0.1942, 0.0614, 0.2284, 0.7694, 0.4195, 0.3797, 0.3607, 0.4739, 0.4429, 0.2802, 0.2529, 0.1332, 0.4537, 0.1205, 0.3059, 0.0323, 0.6229, 0.337, 1, 0.1831, 0.5724, 1, 0.159, 0.7901, 0.5994, 0.3967, 0.3837, 0.1554, 0.0971, 0.1102, 0.0876, 0.2744, 0.3586, 0.0002, 0.8103, 0.0031, 0.0001, 0.4601, 0.3488), method="BY")

## [1] 0.63162168 1.00000000 1.00000000 1.00000000 1.00000000 1.00000000
## [7] 1.00000000 1.00000000 1.00000000 1.00000000 0.01684324 1.00000000
## [13] 1.00000000 1.00000000 1.00000000 1.00000000 1.00000000 1.00000000
## [19] 1.00000000 1.00000000 1.00000000 1.00000000 1.00000000 1.00000000
## [25] 1.00000000 1.00000000 1.00000000 1.00000000 1.00000000 1.00000000
## [31] 1.00000000 1.00000000 1.00000000 1.00000000 1.00000000 1.00000000
## [37] 1.00000000 1.00000000 1.00000000 1.00000000 1.00000000 1.00000000
## [43] 1.00000000 1.00000000 1.00000000 1.00000000 1.00000000 1.00000000
## [49] 1.00000000 0.01684324 1.00000000 0.19580272 0.01684324 1.00000000
## [55] 1.00000000

## Analysis Wahlund effect test for CPB with 11 loci

# Wahlund effect test for CPB with 11 loci
wahlund <- read.csv("CPB_11loci_Wahlund.csv",sep=",", row.names=NULL)
wahlund

## Loci Ht nLDsig
## 1 Cpb14 0.626 1
## 2 Cpb55 0.792 3
## 3 Cpb54 0.889 1
## 4 Cpb62 0.867 1
## 5 Cpb84 0.896 0
## 6 Cpb122 0.877 1
## 7 Cpb112 0.847 0
## 8 Cpb135 0.861 2
## 9 Cpb160 0.859 3
## 10 Cpb139 0.737 1
## 11 Cpb190 0.850 1

with(wahlund, cor.test(Ht, nLDsig, alternative="less", method="spearman"))

## Warning in cor.test.default(Ht, nLDsig, alternative = "less", method =
## "spearman"): Cannot compute exact p-value with ties

##
## Spearman's rank correlation rho
##
## data: Ht and nLDsig
## S = 271.53, p-value = 0.2441
## alternative hypothesis: true rho is less than 0
## sample estimates:
## rho
## -0.23424

## Analysis CPB 11 loci; ajusted p value for observed heterozygocity

p.adjust(c(0.3627, 0.9999, 0.924, 1, 0.9971, 0.9987, 0.9935, 1, 0.1311, 0.9926, 0.9934), method="BH")

## [1] 1 1 1 1 1 1 1 1 1 1 1

## Analysis the correlation betweel FIS and FST and number of blanks for CPB 11 loci and Fis and number of blanks with 7 loci

# Correlation between Fis and fst for cPB with 11 loci

fisfst <- read.csv("cpb_11loci_fis_fst.csv",sep=",", row.names=NULL)
fisfst

## Loci FIS FST
## 1 Cpb14 0.313 -0.002
## 2 Cpb55 0.184 0.061
## 3 Cpb54 -0.118 0.032
## 4 Cpb62 -0.116 0.015
## 5 Cpb84 0.087 0.023
## 6 Cpb122 -0.002 0.048
## 7 Cpb112 0.063 0.029
## 8 Cpb135 0.589 0.066
## 9 Cpb160 0.335 0.056
## 10 Cpb139 -0.299 0.020
## 11 Cpb190 0.285 0.105

with(fisfst, cor.test(FIS, FST, alternative="greater", method="spearman"))

##
## Spearman's rank correlation rho
##
## data: FIS and FST
## S = 116, p-value = 0.0728
## alternative hypothesis: true rho is greater than 0
## sample estimates:
## rho
## 0.4727273

# Correlation between Fis and number of blank for cPB with 11 loci

fisblank11 <- read.csv("Fis_blanks_11loci.csv",sep=",", row.names=NULL)
fisblank11

## Loci Blanks FIS
## 1 Cpb14 0 0.313
## 2 Cpb55 1 0.184
## 3 Cpb54 0 -0.118
## 4 Cpb62 2 -0.116
## 5 Cpb84 0 0.087
## 6 Cpb122 9 -0.002
## 7 Cpb112 0 0.063
## 8 Cpb135 1 0.589
## 9 Cpb160 1 0.335
## 10 Cpb139 1 -0.299
## 11 Cpb190 1 0.285

with(fisblank11, cor.test(FIS, Blanks, alternative="greater", method="spearman"))

## Warning in cor.test.default(FIS, Blanks, alternative = "greater", method =
## "spearman"): Cannot compute exact p-value with ties

##
## Spearman's rank correlation rho
##
## data: FIS and Blanks
## S = 237.22, p-value = 0.5905
## alternative hypothesis: true rho is greater than 0
## sample estimates:
## rho
## -0.07825856

# Correlation between Fis and number of blank for cPB with 7 loci
fisblank7 <- read.csv("Fis_blanks_7loci.csv",sep=",", row.names=NULL)
fisblank7

## Loci Blanks FIS
## 1 Cpb54 0 -0.118
## 2 Cpb62 2 -0.116
## 3 Cpb84 0 0.087
## 4 Cpb122 9 -0.002
## 5 Cpb112 0 0.063
## 6 Cpb160 1 0.335
## 7 Cpb190 1 0.285

with(fisblank7, cor.test(FIS, Blanks, alternative="greater", method="spearman"))

## Warning in cor.test.default(FIS, Blanks, alternative = "greater", method =
## "spearman"): Cannot compute exact p-value with ties

##
## Spearman's rank correlation rho
##
## data: FIS and Blanks
## S = 57.048, p-value = 0.5159
## alternative hypothesis: true rho is greater than 0
## sample estimates:
## rho
## -0.01871203

## Analysis for short allel dominance (SAD) for CPB with 11 loci

SAD <- read.csv("SAD.csv",sep=",", row.names=NULL)
SAD

## loci Allele Capf Theta Smallf p weight
## 1 Cpb14 133 0.000 0.000 0.000 0.007 0.006951
## 2 Cpb14 234 0.000 0.043 -0.045 0.021 0.020559
## 3 Cpb14 236 0.398 -0.026 0.413 0.590 0.241900
## 4 Cpb14 239 -0.030 -0.003 -0.027 0.035 0.033775
## 5 Cpb14 242 0.133 0.040 0.096 0.076 0.070224
## 6 Cpb14 243 0.662 -0.014 0.667 0.021 0.020559
## 7 Cpb14 245 0.286 0.019 0.273 0.146 0.124684
## 8 Cpb14 246 1.000 0.000 1.000 0.014 0.013804
## 9 Cpb14 248 -0.034 -0.016 -0.018 0.035 0.033775
## 10 Cpb14 250 1.000 0.000 1.000 0.014 0.013804
## 11 Cpb14 256 -0.014 0.001 -0.015 0.021 0.020559
## 12 Cpb14 258 1.000 0.000 1.000 0.014 0.013804
## 13 Cpb14 261 0.000 0.000 0.000 0.007 0.006951
## 14 Cpb55 254 -0.018 0.035 -0.055 0.035 0.033775
## 15 Cpb55 256 0.000 0.001 -0.001 0.007 0.006951
## 16 Cpb55 260 0.233 0.112 0.137 0.352 0.228096
## 17 Cpb55 262 0.127 0.007 0.121 0.162 0.135756
## 18 Cpb55 265 0.341 0.050 0.307 0.211 0.166479
## 19 Cpb55 268 -0.027 -0.016 -0.011 0.028 0.027216
## 20 Cpb55 271 0.313 0.049 0.278 0.113 0.100231
## 21 Cpb55 274 0.249 0.145 0.121 0.056 0.052864
## 22 Cpb55 276 0.000 0.000 0.000 0.007 0.006951
## 23 Cpb55 280 0.489 -0.011 0.495 0.028 0.027216
## 24 Cpb54 131 0.000 0.000 0.000 0.007 0.006951
## 25 Cpb54 166 0.000 0.000 0.000 0.007 0.006951
## 26 Cpb54 249 0.000 0.043 -0.045 0.021 0.020559
## 27 Cpb54 253 -0.017 0.036 -0.055 0.035 0.033775
## 28 Cpb54 255 0.000 0.000 0.000 0.007 0.006951
## 29 Cpb54 257 -0.070 0.086 -0.171 0.097 0.087591
## 30 Cpb54 259 -0.268 0.026 -0.303 0.222 0.172716
## 31 Cpb54 261 -0.025 0.039 -0.067 0.139 0.119679
## 32 Cpb54 263 0.662 -0.014 0.667 0.021 0.020559
## 33 Cpb54 265 -0.100 0.003 -0.103 0.097 0.087591
## 34 Cpb54 267 -0.029 0.024 -0.053 0.042 0.040236
## 35 Cpb54 269 -0.129 0.044 -0.181 0.132 0.114576
## 36 Cpb54 271 -0.055 0.037 -0.095 0.069 0.064239
## 37 Cpb54 273 -0.017 0.036 -0.055 0.035 0.033775
## 38 Cpb54 275 -0.011 -0.011 0.000 0.014 0.013804
## 39 Cpb54 277 0.000 0.000 0.000 0.007 0.006951
## 40 Cpb54 279 0.000 0.022 -0.022 0.014 0.013804
## 41 Cpb54 281 -0.030 -0.003 -0.027 0.035 0.033775
## 42 Cpb52 185 0.000 -0.001 0.001 0.007 0.006951
## 43 Cpb52 205 0.001 0.002 -0.001 0.007 0.006951
## 44 Cpb52 217 -0.043 0.006 -0.050 0.050 0.047500
## 45 Cpb52 219 -0.233 0.027 -0.266 0.200 0.160000
## 46 Cpb52 221 -0.092 -0.014 -0.076 0.086 0.078604
## 47 Cpb52 223 -0.061 0.009 -0.070 0.200 0.160000
## 48 Cpb52 225 -0.061 -0.012 -0.049 0.150 0.127500
## 49 Cpb52 227 -0.053 0.048 -0.106 0.071 0.065959
## 50 Cpb52 228 -0.040 -0.007 -0.032 0.043 0.041151
## 51 Cpb52 231 -0.128 0.059 -0.198 0.136 0.117504
## 52 Cpb52 233 -0.015 0.000 -0.015 0.021 0.020559
## 53 Cpb52 237 -0.015 -0.001 -0.014 0.021 0.020559
## 54 Cpb52 257 0.000 -0.001 0.001 0.007 0.006951
## 55 Cpb84 197 0.000 0.000 0.000 0.007 0.006951
## 56 Cpb84 203 -0.014 0.001 -0.015 0.021 0.020559
## 57 Cpb84 205 0.000 0.000 0.000 0.007 0.006951
## 58 Cpb84 207 -0.026 0.010 -0.036 0.035 0.033775
## 59 Cpb84 209 0.000 0.000 0.000 0.007 0.006951
## 60 Cpb84 211 0.432 0.139 0.341 0.118 0.104076
## 61 Cpb84 213 0.184 0.027 0.161 0.063 0.059031
## 62 Cpb84 215 0.146 0.008 0.139 0.215 0.168775
## 63 Cpb84 217 0.178 0.005 0.174 0.063 0.059031
## 64 Cpb84 219 0.025 0.006 0.018 0.111 0.098679
## 65 Cpb84 221 -0.080 -0.011 -0.068 0.076 0.070224
## 66 Cpb84 223 0.000 0.000 0.000 0.007 0.006951
## 67 Cpb84 225 -0.021 0.001 -0.022 0.028 0.027216
## 68 Cpb84 227 -0.026 0.010 -0.036 0.035 0.033775
## 69 Cpb84 229 0.000 0.022 -0.022 0.014 0.013804
## 70 Cpb84 231 -0.014 0.001 -0.015 0.021 0.020559
## 71 Cpb84 233 -0.014 0.001 -0.015 0.021 0.020559
## 72 Cpb84 235 0.080 0.008 0.073 0.139 0.119679
## 73 Cpb84 245 0.000 0.022 -0.022 0.014 0.013804
## 74 Cpb122 184 -0.014 -0.016 0.003 0.016 0.015744
## 75 Cpb122 186 -0.041 0.028 -0.071 0.056 0.052864
## 76 Cpb122 188 0.300 -0.030 0.321 0.048 0.045696
## 77 Cpb122 190 -0.018 -0.005 -0.013 0.024 0.023424
## 78 Cpb122 192 -0.003 0.063 -0.071 0.032 0.030976
## 79 Cpb122 194 -0.041 0.003 -0.044 0.048 0.045696
## 80 Cpb122 196 0.004 -0.005 0.009 0.127 0.110871
## 81 Cpb122 198 -0.095 0.139 -0.272 0.135 0.116775
## 82 Cpb122 200 0.244 -0.029 0.265 0.056 0.052864
## 83 Cpb122 202 0.082 -0.016 0.096 0.095 0.085975
## 84 Cpb122 204 0.127 0.134 -0.008 0.254 0.189484
## 85 Cpb122 206 -0.011 -0.009 -0.002 0.016 0.015744
## 86 Cpb122 208 -0.001 -0.003 0.002 0.008 0.007936
## 87 Cpb122 210 -0.009 0.046 -0.057 0.032 0.030976
## 88 Cpb122 212 -0.014 -0.016 0.003 0.016 0.015744
## 89 Cpb122 214 -0.003 0.016 -0.019 0.016 0.015744
## 90 Cpb122 220 -0.011 -0.009 -0.002 0.016 0.015744
## 91 Cpb122 226 -0.001 -0.003 0.002 0.008 0.007936
## 92 Cpb112 184 -0.011 -0.011 0.000 0.014 0.013804
## 93 Cpb112 192 0.000 0.022 -0.022 0.014 0.013804
## 94 Cpb112 200 -0.021 -0.021 0.000 0.021 0.020559
## 95 Cpb112 202 -0.021 -0.021 0.000 0.021 0.020559
## 96 Cpb112 204 -0.014 0.001 -0.015 0.021 0.020559
## 97 Cpb112 208 0.000 0.000 0.000 0.007 0.006951
## 98 Cpb112 216 0.000 0.000 0.000 0.007 0.006951
## 99 Cpb112 230 0.000 0.000 0.000 0.007 0.006951
## 100 Cpb112 296 -0.086 0.088 -0.191 0.111 0.098679
## 101 Cpb112 298 -0.014 0.001 -0.015 0.021 0.020559
## 102 Cpb112 300 0.144 0.058 0.091 0.160 0.134400
## 103 Cpb112 302 0.343 0.016 0.332 0.250 0.187500
## 104 Cpb112 304 0.052 0.030 0.023 0.222 0.172716
## 105 Cpb112 306 -0.073 0.007 -0.081 0.076 0.070224
## 106 Cpb112 308 -0.040 -0.010 -0.030 0.042 0.040236
## 107 Cpb112 310 0.000 0.000 0.000 0.007 0.006951
## 108 Cpb135 212 1.000 0.086 1.000 0.042 0.040236
## 109 Cpb135 216 1.000 0.075 1.000 0.113 0.100231
## 110 Cpb135 218 -0.026 0.010 -0.037 0.035 0.033775
## 111 Cpb135 220 0.348 0.003 0.346 0.183 0.149511
## 112 Cpb135 222 -0.045 -0.014 -0.031 0.141 0.121119
## 113 Cpb135 224 0.943 0.163 0.932 0.134 0.116044
## 114 Cpb135 228 0.825 0.016 0.822 0.197 0.158191
## 115 Cpb135 230 0.741 0.185 0.682 0.148 0.126096
## 116 Cpb135 238 0.000 0.001 -0.001 0.007 0.006951
## 117 Cpb160 264 1.000 0.002 1.000 0.014 0.013804
## 118 Cpb160 266 0.332 0.072 0.281 0.077 0.071071
## 119 Cpb160 268 0.416 0.061 0.378 0.120 0.105600
## 120 Cpb160 270 0.177 0.003 0.175 0.063 0.059031
## 121 Cpb160 272 0.574 0.107 0.523 0.289 0.205479
## 122 Cpb160 274 0.592 0.075 0.559 0.113 0.100231
## 123 Cpb160 278 0.129 0.046 0.088 0.127 0.110871
## 124 Cpb160 280 -0.027 -0.015 -0.012 0.028 0.027216
## 125 Cpb160 282 0.064 0.027 0.039 0.099 0.089199
## 126 Cpb160 284 -0.011 -0.011 0.000 0.014 0.013804
## 127 Cpb160 286 -0.015 0.000 -0.014 0.021 0.020559
## 128 Cpb160 298 1.000 -0.021 1.000 0.028 0.027216
## 129 Cpb160 302 0.000 0.000 0.000 0.007 0.006951
## 130 Cpb139 224 0.000 0.000 0.000 0.007 0.006951
## 131 Cpb139 228 -0.805 -0.002 -0.801 0.479 0.249559
## 132 Cpb139 230 -0.084 0.003 -0.088 0.085 0.077775
## 133 Cpb139 241 0.000 0.000 0.000 0.007 0.006951
## 134 Cpb139 263 0.000 0.000 0.000 0.007 0.006951
## 135 Cpb139 267 -0.019 0.075 -0.102 0.049 0.046599
## 136 Cpb139 268 -0.064 0.037 -0.105 0.077 0.071071
## 137 Cpb139 270 -0.031 -0.004 -0.027 0.035 0.033775
## 138 Cpb139 273 -0.115 0.013 -0.130 0.113 0.100231
## 139 Cpb139 275 0.302 0.099 0.225 0.085 0.077775
## 140 Cpb139 278 -0.015 0.000 -0.014 0.021 0.020559
## 141 Cpb139 280 -0.014 0.002 -0.016 0.021 0.020559
## 142 Cpb139 297 0.000 0.000 0.000 0.007 0.006951
## 143 Cpb190 281 0.000 0.001 -0.001 0.007 0.006951
## 144 Cpb190 283 0.001 0.024 -0.024 0.014 0.013804
## 145 Cpb190 287 0.000 0.000 0.000 0.007 0.006951
## 146 Cpb190 289 0.000 0.000 0.000 0.007 0.006951
## 147 Cpb190 291 0.639 0.062 0.615 0.106 0.094764
## 148 Cpb190 293 0.464 0.130 0.385 0.225 0.174375
## 149 Cpb190 295 0.359 0.175 0.223 0.190 0.153900
## 150 Cpb190 297 0.214 0.038 0.183 0.169 0.140439
## 151 Cpb190 299 0.239 0.179 0.073 0.141 0.121119
## 152 Cpb190 303 0.537 0.046 0.514 0.099 0.089199
## 153 Cpb190 305 -0.014 0.002 -0.016 0.021 0.020559
## 154 Cpb190 307 0.000 0.021 -0.022 0.014 0.013804

SAD$weight<- as.numeric(SAD$weight)
SAD$Allele <- as.numeric(SAD$Allele)

SAD_Cpb14 <- subset(SAD,loci=="Cpb14")
SAD_Cpb14

## loci Allele Capf Theta Smallf p weight
## 1 Cpb14 133 0.000 0.000 0.000 0.007 0.006951
## 2 Cpb14 234 0.000 0.043 -0.045 0.021 0.020559
## 3 Cpb14 236 0.398 -0.026 0.413 0.590 0.241900
## 4 Cpb14 239 -0.030 -0.003 -0.027 0.035 0.033775
## 5 Cpb14 242 0.133 0.040 0.096 0.076 0.070224
## 6 Cpb14 243 0.662 -0.014 0.667 0.021 0.020559
## 7 Cpb14 245 0.286 0.019 0.273 0.146 0.124684
## 8 Cpb14 246 1.000 0.000 1.000 0.014 0.013804
## 9 Cpb14 248 -0.034 -0.016 -0.018 0.035 0.033775
## 10 Cpb14 250 1.000 0.000 1.000 0.014 0.013804
## 11 Cpb14 256 -0.014 0.001 -0.015 0.021 0.020559
## 12 Cpb14 258 1.000 0.000 1.000 0.014 0.013804
## 13 Cpb14 261 0.000 0.000 0.000 0.007 0.006951

with(SAD_Cpb14, cor.test(Allele, Capf, alternative="less", method="spearman"))

## Warning in cor.test.default(Allele, Capf, alternative = "less", method =
## "spearman"): Cannot compute exact p-value with ties

##
## Spearman's rank correlation rho
##
## data: Allele and Capf
## S = 301.31, p-value = 0.7132
## alternative hypothesis: true rho is less than 0
## sample estimates:
## rho
## 0.1722329

SAD_Cpb55 <- subset(SAD,loci=="Cpb55")
SAD_Cpb55

## loci Allele Capf Theta Smallf p weight
## 14 Cpb55 254 -0.018 0.035 -0.055 0.035 0.033775
## 15 Cpb55 256 0.000 0.001 -0.001 0.007 0.006951
## 16 Cpb55 260 0.233 0.112 0.137 0.352 0.228096
## 17 Cpb55 262 0.127 0.007 0.121 0.162 0.135756
## 18 Cpb55 265 0.341 0.050 0.307 0.211 0.166479
## 19 Cpb55 268 -0.027 -0.016 -0.011 0.028 0.027216
## 20 Cpb55 271 0.313 0.049 0.278 0.113 0.100231
## 21 Cpb55 274 0.249 0.145 0.121 0.056 0.052864
## 22 Cpb55 276 0.000 0.000 0.000 0.007 0.006951
## 23 Cpb55 280 0.489 -0.011 0.495 0.028 0.027216

with(SAD_Cpb55, cor.test(Allele, Capf, alternative="less", method="spearman"))

## Warning in cor.test.default(Allele, Capf, alternative = "less", method =
## "spearman"): Cannot compute exact p-value with ties

##
## Spearman's rank correlation rho
##
## data: Allele and Capf
## S = 86.763, p-value = 0.9169
## alternative hypothesis: true rho is less than 0
## sample estimates:
## rho
## 0.4741663

SAD_Cpb54 <- subset(SAD,loci=="Cpb54")
SAD_Cpb54

## loci Allele Capf Theta Smallf p weight
## 24 Cpb54 131 0.000 0.000 0.000 0.007 0.006951
## 25 Cpb54 166 0.000 0.000 0.000 0.007 0.006951
## 26 Cpb54 249 0.000 0.043 -0.045 0.021 0.020559
## 27 Cpb54 253 -0.017 0.036 -0.055 0.035 0.033775
## 28 Cpb54 255 0.000 0.000 0.000 0.007 0.006951
## 29 Cpb54 257 -0.070 0.086 -0.171 0.097 0.087591
## 30 Cpb54 259 -0.268 0.026 -0.303 0.222 0.172716
## 31 Cpb54 261 -0.025 0.039 -0.067 0.139 0.119679
## 32 Cpb54 263 0.662 -0.014 0.667 0.021 0.020559
## 33 Cpb54 265 -0.100 0.003 -0.103 0.097 0.087591
## 34 Cpb54 267 -0.029 0.024 -0.053 0.042 0.040236
## 35 Cpb54 269 -0.129 0.044 -0.181 0.132 0.114576
## 36 Cpb54 271 -0.055 0.037 -0.095 0.069 0.064239
## 37 Cpb54 273 -0.017 0.036 -0.055 0.035 0.033775
## 38 Cpb54 275 -0.011 -0.011 0.000 0.014 0.013804
## 39 Cpb54 277 0.000 0.000 0.000 0.007 0.006951
## 40 Cpb54 279 0.000 0.022 -0.022 0.014 0.013804
## 41 Cpb54 281 -0.030 -0.003 -0.027 0.035 0.033775

with(SAD_Cpb54, cor.test(Allele, Capf, alternative="less", method="spearman"))

## Warning in cor.test.default(Allele, Capf, alternative = "less", method =
## "spearman"): Cannot compute exact p-value with ties

##
## Spearman's rank correlation rho
##
## data: Allele and Capf
## S = 1151.4, p-value = 0.2272
## alternative hypothesis: true rho is less than 0
## sample estimates:
## rho
## -0.1882566

lm_SAD_54 <- lm(Smallf ~ Allele, data=SAD_Cpb54, weights=weight)
summary(lm_SAD_54)

##
## Call:
## lm(formula = Smallf ~ Allele, data = SAD_Cpb54, weights = weight)
##
## Weighted Residuals:
## Min 1Q Median 3Q Max
## -0.076276 0.006953 0.010745 0.012895 0.112726
##
## Coefficients:
## Estimate Std. Error t value Pr(>|t|)
## (Intercept) -1.377e-01 6.293e-01 -0.219 0.830
## Allele 7.054e-05 2.396e-03 0.029 0.977
##
## Residual standard error: 0.03638 on 16 degrees of freedom
## Multiple R-squared: 5.417e-05, Adjusted R-squared: -0.06244
## F-statistic: 0.0008667 on 1 and 16 DF, p-value: 0.9769

SAD_Cpb52 <- subset(SAD,loci=="Cpb52")
SAD_Cpb52

## loci Allele Capf Theta Smallf p weight
## 42 Cpb52 185 0.000 -0.001 0.001 0.007 0.006951
## 43 Cpb52 205 0.001 0.002 -0.001 0.007 0.006951
## 44 Cpb52 217 -0.043 0.006 -0.050 0.050 0.047500
## 45 Cpb52 219 -0.233 0.027 -0.266 0.200 0.160000
## 46 Cpb52 221 -0.092 -0.014 -0.076 0.086 0.078604
## 47 Cpb52 223 -0.061 0.009 -0.070 0.200 0.160000
## 48 Cpb52 225 -0.061 -0.012 -0.049 0.150 0.127500
## 49 Cpb52 227 -0.053 0.048 -0.106 0.071 0.065959
## 50 Cpb52 228 -0.040 -0.007 -0.032 0.043 0.041151
## 51 Cpb52 231 -0.128 0.059 -0.198 0.136 0.117504
## 52 Cpb52 233 -0.015 0.000 -0.015 0.021 0.020559
## 53 Cpb52 237 -0.015 -0.001 -0.014 0.021 0.020559
## 54 Cpb52 257 0.000 -0.001 0.001 0.007 0.006951

with(SAD_Cpb52, cor.test(Allele, Capf, alternative="less", method="spearman"))

## Warning in cor.test.default(Allele, Capf, alternative = "less", method =
## "spearman"): Cannot compute exact p-value with ties

##
## Spearman's rank correlation rho
##
## data: Allele and Capf
## S = 349.94, p-value = 0.5498
## alternative hypothesis: true rho is less than 0
## sample estimates:
## rho
## 0.03862102

SAD_Cpb84 <- subset(SAD,loci=="Cpb84")
SAD_Cpb84

## loci Allele Capf Theta Smallf p weight
## 55 Cpb84 197 0.000 0.000 0.000 0.007 0.006951
## 56 Cpb84 203 -0.014 0.001 -0.015 0.021 0.020559
## 57 Cpb84 205 0.000 0.000 0.000 0.007 0.006951
## 58 Cpb84 207 -0.026 0.010 -0.036 0.035 0.033775
## 59 Cpb84 209 0.000 0.000 0.000 0.007 0.006951
## 60 Cpb84 211 0.432 0.139 0.341 0.118 0.104076
## 61 Cpb84 213 0.184 0.027 0.161 0.063 0.059031
## 62 Cpb84 215 0.146 0.008 0.139 0.215 0.168775
## 63 Cpb84 217 0.178 0.005 0.174 0.063 0.059031
## 64 Cpb84 219 0.025 0.006 0.018 0.111 0.098679
## 65 Cpb84 221 -0.080 -0.011 -0.068 0.076 0.070224
## 66 Cpb84 223 0.000 0.000 0.000 0.007 0.006951
## 67 Cpb84 225 -0.021 0.001 -0.022 0.028 0.027216
## 68 Cpb84 227 -0.026 0.010 -0.036 0.035 0.033775
## 69 Cpb84 229 0.000 0.022 -0.022 0.014 0.013804
## 70 Cpb84 231 -0.014 0.001 -0.015 0.021 0.020559
## 71 Cpb84 233 -0.014 0.001 -0.015 0.021 0.020559
## 72 Cpb84 235 0.080 0.008 0.073 0.139 0.119679
## 73 Cpb84 245 0.000 0.022 -0.022 0.014 0.013804

with(SAD_Cpb84, cor.test(Allele, Capf, alternative="less", method="spearman"))

## Warning in cor.test.default(Allele, Capf, alternative = "less", method =
## "spearman"): Cannot compute exact p-value with ties

##
## Spearman's rank correlation rho
##
## data: Allele and Capf
## S = 1289.6, p-value = 0.2961
## alternative hypothesis: true rho is less than 0
## sample estimates:
## rho
## -0.1312709

SAD_Cpb84 <- subset(SAD,loci=="Cpb84")
SAD_Cpb84

## loci Allele Capf Theta Smallf p weight
## 55 Cpb84 197 0.000 0.000 0.000 0.007 0.006951
## 56 Cpb84 203 -0.014 0.001 -0.015 0.021 0.020559
## 57 Cpb84 205 0.000 0.000 0.000 0.007 0.006951
## 58 Cpb84 207 -0.026 0.010 -0.036 0.035 0.033775
## 59 Cpb84 209 0.000 0.000 0.000 0.007 0.006951
## 60 Cpb84 211 0.432 0.139 0.341 0.118 0.104076
## 61 Cpb84 213 0.184 0.027 0.161 0.063 0.059031
## 62 Cpb84 215 0.146 0.008 0.139 0.215 0.168775
## 63 Cpb84 217 0.178 0.005 0.174 0.063 0.059031
## 64 Cpb84 219 0.025 0.006 0.018 0.111 0.098679
## 65 Cpb84 221 -0.080 -0.011 -0.068 0.076 0.070224
## 66 Cpb84 223 0.000 0.000 0.000 0.007 0.006951
## 67 Cpb84 225 -0.021 0.001 -0.022 0.028 0.027216
## 68 Cpb84 227 -0.026 0.010 -0.036 0.035 0.033775
## 69 Cpb84 229 0.000 0.022 -0.022 0.014 0.013804
## 70 Cpb84 231 -0.014 0.001 -0.015 0.021 0.020559
## 71 Cpb84 233 -0.014 0.001 -0.015 0.021 0.020559
## 72 Cpb84 235 0.080 0.008 0.073 0.139 0.119679
## 73 Cpb84 245 0.000 0.022 -0.022 0.014 0.013804

with(SAD_Cpb84, cor.test(Allele, Capf, alternative="less", method="spearman"))

## Warning in cor.test.default(Allele, Capf, alternative = "less", method =
## "spearman"): Cannot compute exact p-value with ties

##
## Spearman's rank correlation rho
##
## data: Allele and Capf
## S = 1289.6, p-value = 0.2961
## alternative hypothesis: true rho is less than 0
## sample estimates:
## rho
## -0.1312709

lm_SAD_84 <- lm(Smallf ~ Allele, data=SAD_Cpb84, weights=weight)
summary(lm_SAD_84)

##
## Call:
## lm(formula = Smallf ~ Allele, data = SAD_Cpb84, weights = weight)
##
## Weighted Residuals:
## Min 1Q Median 3Q Max
## -0.039938 -0.016543 -0.007726 0.005493 0.067618
##
## Coefficients:
## Estimate Std. Error t value Pr(>|t|)
## (Intercept) 1.158796 0.623016 1.860 0.0803 .
## Allele -0.004869 0.002835 -1.718 0.1040
## ---
## Signif. codes: 0 '***' 0.001 '**' 0.01 '*' 0.05 '.' 0.1 ' ' 1
##
## Residual standard error: 0.02517 on 17 degrees of freedom
## Multiple R-squared: 0.1479, Adjusted R-squared: 0.09776
## F-statistic: 2.95 on 1 and 17 DF, p-value: 0.104

SAD_Cpb122 <- subset(SAD,loci=="Cpb122")
SAD_Cpb122

## loci Allele Capf Theta Smallf p weight
## 74 Cpb122 184 -0.014 -0.016 0.003 0.016 0.015744
## 75 Cpb122 186 -0.041 0.028 -0.071 0.056 0.052864
## 76 Cpb122 188 0.300 -0.030 0.321 0.048 0.045696
## 77 Cpb122 190 -0.018 -0.005 -0.013 0.024 0.023424
## 78 Cpb122 192 -0.003 0.063 -0.071 0.032 0.030976
## 79 Cpb122 194 -0.041 0.003 -0.044 0.048 0.045696
## 80 Cpb122 196 0.004 -0.005 0.009 0.127 0.110871
## 81 Cpb122 198 -0.095 0.139 -0.272 0.135 0.116775
## 82 Cpb122 200 0.244 -0.029 0.265 0.056 0.052864
## 83 Cpb122 202 0.082 -0.016 0.096 0.095 0.085975
## 84 Cpb122 204 0.127 0.134 -0.008 0.254 0.189484
## 85 Cpb122 206 -0.011 -0.009 -0.002 0.016 0.015744
## 86 Cpb122 208 -0.001 -0.003 0.002 0.008 0.007936
## 87 Cpb122 210 -0.009 0.046 -0.057 0.032 0.030976
## 88 Cpb122 212 -0.014 -0.016 0.003 0.016 0.015744
## 89 Cpb122 214 -0.003 0.016 -0.019 0.016 0.015744
## 90 Cpb122 220 -0.011 -0.009 -0.002 0.016 0.015744
## 91 Cpb122 226 -0.001 -0.003 0.002 0.008 0.007936

with(SAD_Cpb122, cor.test(Allele, Capf, alternative="less", method="spearman"))

## Warning in cor.test.default(Allele, Capf, alternative = "less", method =
## "spearman"): Cannot compute exact p-value with ties

##
## Spearman's rank correlation rho
##
## data: Allele and Capf
## S = 788.53, p-value = 0.7703
## alternative hypothesis: true rho is less than 0
## sample estimates:
## rho
## 0.1862396

SAD_Cpb135 <- subset(SAD,loci=="Cpb135")
SAD_Cpb135

## loci Allele Capf Theta Smallf p weight
## 108 Cpb135 212 1.000 0.086 1.000 0.042 0.040236
## 109 Cpb135 216 1.000 0.075 1.000 0.113 0.100231
## 110 Cpb135 218 -0.026 0.010 -0.037 0.035 0.033775
## 111 Cpb135 220 0.348 0.003 0.346 0.183 0.149511
## 112 Cpb135 222 -0.045 -0.014 -0.031 0.141 0.121119
## 113 Cpb135 224 0.943 0.163 0.932 0.134 0.116044
## 114 Cpb135 228 0.825 0.016 0.822 0.197 0.158191
## 115 Cpb135 230 0.741 0.185 0.682 0.148 0.126096
## 116 Cpb135 238 0.000 0.001 -0.001 0.007 0.006951

with(SAD_Cpb135, cor.test(Allele, Capf, alternative="less", method="spearman"))

## Warning in cor.test.default(Allele, Capf, alternative = "less", method =
## "spearman"): Cannot compute exact p-value with ties

##
## Spearman's rank correlation rho
##
## data: Allele and Capf
## S = 163.18, p-value = 0.1708
## alternative hypothesis: true rho is less than 0
## sample estimates:
## rho
## -0.3598358

lm_SAD_135 <- lm(Smallf ~ Allele, data=SAD_Cpb135, weights=weight)
summary(lm_SAD_135)

##
## Call:
## lm(formula = Smallf ~ Allele, data = SAD_Cpb135, weights = weight)
##
## Weighted Residuals:
## Min 1Q Median 3Q Max
## -0.21869 -0.09513 0.02244 0.08614 0.13256
##
## Coefficients:
## Estimate Std. Error t value Pr(>|t|)
## (Intercept) 0.002308 5.919472 0.000 1.000
## Allele 0.002680 0.026536 0.101 0.922
##
## Residual standard error: 0.1297 on 7 degrees of freedom
## Multiple R-squared: 0.001456, Adjusted R-squared: -0.1412
## F-statistic: 0.0102 on 1 and 7 DF, p-value: 0.9224

SAD_Cpb160 <- subset(SAD,loci=="Cpb160")
SAD_Cpb160

## loci Allele Capf Theta Smallf p weight
## 117 Cpb160 264 1.000 0.002 1.000 0.014 0.013804
## 118 Cpb160 266 0.332 0.072 0.281 0.077 0.071071
## 119 Cpb160 268 0.416 0.061 0.378 0.120 0.105600
## 120 Cpb160 270 0.177 0.003 0.175 0.063 0.059031
## 121 Cpb160 272 0.574 0.107 0.523 0.289 0.205479
## 122 Cpb160 274 0.592 0.075 0.559 0.113 0.100231
## 123 Cpb160 278 0.129 0.046 0.088 0.127 0.110871
## 124 Cpb160 280 -0.027 -0.015 -0.012 0.028 0.027216
## 125 Cpb160 282 0.064 0.027 0.039 0.099 0.089199
## 126 Cpb160 284 -0.011 -0.011 0.000 0.014 0.013804
## 127 Cpb160 286 -0.015 0.000 -0.014 0.021 0.020559
## 128 Cpb160 298 1.000 -0.021 1.000 0.028 0.027216
## 129 Cpb160 302 0.000 0.000 0.000 0.007 0.006951

with(SAD_Cpb160, cor.test(Allele, Capf, alternative="less", method="spearman"))

## Warning in cor.test.default(Allele, Capf, alternative = "less", method =
## "spearman"): Cannot compute exact p-value with ties

##
## Spearman's rank correlation rho
##
## data: Allele and Capf
## S = 541.24, p-value = 0.04575
## alternative hypothesis: true rho is less than 0
## sample estimates:
## rho
## -0.4869331

lm_SAD_160 <- lm(Smallf ~ Allele, data=SAD_Cpb160, weights=weight)
summary(lm_SAD_160)

##
## Call:
## lm(formula = Smallf ~ Allele, data = SAD_Cpb160, weights = weight)
##
## Weighted Residuals:
## Min 1Q Median 3Q Max
## -0.07647 -0.04631 -0.02894 0.06882 0.13234
##
## Coefficients:
## Estimate Std. Error t value Pr(>|t|)
## (Intercept) 1.983341 2.852520 0.695 0.501
## Allele -0.005992 0.010382 -0.577 0.575
##
## Residual standard error: 0.06968 on 11 degrees of freedom
## Multiple R-squared: 0.02939, Adjusted R-squared: -0.05885
## F-statistic: 0.3331 on 1 and 11 DF, p-value: 0.5755

SAD_Cpb139 <- subset(SAD,loci=="Cpb139")
SAD_Cpb139

## loci Allele Capf Theta Smallf p weight
## 130 Cpb139 224 0.000 0.000 0.000 0.007 0.006951
## 131 Cpb139 228 -0.805 -0.002 -0.801 0.479 0.249559
## 132 Cpb139 230 -0.084 0.003 -0.088 0.085 0.077775
## 133 Cpb139 241 0.000 0.000 0.000 0.007 0.006951
## 134 Cpb139 263 0.000 0.000 0.000 0.007 0.006951
## 135 Cpb139 267 -0.019 0.075 -0.102 0.049 0.046599
## 136 Cpb139 268 -0.064 0.037 -0.105 0.077 0.071071
## 137 Cpb139 270 -0.031 -0.004 -0.027 0.035 0.033775
## 138 Cpb139 273 -0.115 0.013 -0.130 0.113 0.100231
## 139 Cpb139 275 0.302 0.099 0.225 0.085 0.077775
## 140 Cpb139 278 -0.015 0.000 -0.014 0.021 0.020559
## 141 Cpb139 280 -0.014 0.002 -0.016 0.021 0.020559
## 142 Cpb139 297 0.000 0.000 0.000 0.007 0.006951

## Analysis the bionomial test of the expected blank for null alleles of CPB with 11 loci

stut <- read.csv("cpb_11loci_stutt_bionom.csv",sep=",", row.names=NULL)
stut

## Loci N NExpBlanks ObsBlanks f.ExpBlanks.
## 1 Cpb14 72 1.0236666580 0 0.0142175920
## 2 Cpb55 72 0.9190134340 1 0.0127640750
## 3 Cpb54 72 0.0019053140 0 0.0000265000
## 4 Cpb62 72 0.2236329820 2 0.0031060140
## 5 Cpb84 72 0.0560796310 0 0.0007788840
## 6 Cpb122 72 3.6207623160 9 0.0502883660
## 7 Cpb112 72 0.0000000072 0 0.0000000001
## 8 Cpb135 72 5.4324042340 1 0.0754500590
## 9 Cpb160 72 2.2479744580 1 0.0312218670
## 10 Cpb139 72 0.0825557400 1 0.0011466080
## 11 Cpb190 72 1.4180291060 1 0.0196948490

stut_cpb14<-subset(stut,Loci=="Cpb14")
stut_cpb14

## Loci N NExpBlanks ObsBlanks f.ExpBlanks.
## 1 Cpb14 72 1.023667 0 0.01421759

with(stut_cpb14,binom.test(ObsBlanks, N, f.ExpBlanks., alternative="less"))

##
## Exact binomial test
##
## data: ObsBlanks and N
## number of successes = 0, number of trials = 72, p-value = 0.3566
## alternative hypothesis: true probability of success is less than 0.01421759
## 95 percent confidence interval:
## 0.00000000 0.04075369
## sample estimates:
## probability of success
## 0

stut_cpb55<-subset(stut,Loci=="Cpb55")
stut_cpb55

## Loci N NExpBlanks ObsBlanks f.ExpBlanks.
## 2 Cpb55 72 0.9190134 1 0.01276407

with(stut_cpb55,binom.test(ObsBlanks, N, f.ExpBlanks., alternative="less"))

##
## Exact binomial test
##
## data: ObsBlanks and N
## number of successes = 1, number of trials = 72, p-value = 0.7657
## alternative hypothesis: true probability of success is less than 0.01276407
## 95 percent confidence interval:
## 0.00000000 0.06419854
## sample estimates:
## probability of success
## 0.01388889

stut_cpb54<-subset(stut,Loci=="Cpb54")
stut_cpb54

## Loci N NExpBlanks ObsBlanks f.ExpBlanks.
## 3 Cpb54 72 0.001905314 0 2.65e-05

with(stut_cpb54,binom.test(ObsBlanks, N, f.ExpBlanks., alternative="less"))

##
## Exact binomial test
##
## data: ObsBlanks and N
## number of successes = 0, number of trials = 72, p-value = 0.9981
## alternative hypothesis: true probability of success is less than 2.65e-05
## 95 percent confidence interval:
## 0.00000000 0.04075369
## sample estimates:
## probability of success
## 0

stut_cpb62<-subset(stut,Loci=="Cpb62")
stut_cpb62

## Loci N NExpBlanks ObsBlanks f.ExpBlanks.
## 4 Cpb62 72 0.223633 2 0.003106014

with(stut_cpb62,binom.test(ObsBlanks, N, f.ExpBlanks., alternative="less"))

##
## Exact binomial test
##
## data: ObsBlanks and N
## number of successes = 2, number of trials = 72, p-value = 0.9985
## alternative hypothesis: true probability of success is less than 0.003106014
## 95 percent confidence interval:
## 0.00000000 0.08486917
## sample estimates:
## probability of success
## 0.02777778

stut_cpb84<-subset(stut,Loci=="Cpb84")
stut_cpb84

## Loci N NExpBlanks ObsBlanks f.ExpBlanks.
## 5 Cpb84 72 0.05607963 0 0.000778884

with(stut_cpb84,binom.test(ObsBlanks, N, f.ExpBlanks., alternative="less"))

##
## Exact binomial test
##
## data: ObsBlanks and N
## number of successes = 0, number of trials = 72, p-value = 0.9454
## alternative hypothesis: true probability of success is less than 0.000778884
## 95 percent confidence interval:
## 0.00000000 0.04075369
## sample estimates:
## probability of success
## 0

stut_cpb122<-subset(stut,Loci=="Cpb122")
stut_cpb122

## Loci N NExpBlanks ObsBlanks f.ExpBlanks.
## 6 Cpb122 72 3.620762 9 0.05028837

with(stut_cpb122,binom.test(ObsBlanks, N, f.ExpBlanks., alternative="less"))

##
## Exact binomial test
##
## data: ObsBlanks and N
## number of successes = 9, number of trials = 72, p-value = 0.9968
## alternative hypothesis: true probability of success is less than 0.05028837
## 95 percent confidence interval:
## 0.0000000 0.2079915
## sample estimates:
## probability of success
## 0.125

stut_cpb112<-subset(stut,Loci=="Cpb112")
stut_cpb112

## Loci N NExpBlanks ObsBlanks f.ExpBlanks.
## 7 Cpb112 72 7.2e-09 0 1e-10

with(stut_cpb112,binom.test(ObsBlanks, N, f.ExpBlanks., alternative="less"))

##
## Exact binomial test
##
## data: ObsBlanks and N
## number of successes = 0, number of trials = 72, p-value = 1
## alternative hypothesis: true probability of success is less than 1e-10
## 95 percent confidence interval:
## 0.00000000 0.04075369
## sample estimates:
## probability of success
## 0

stut_cpb135<-subset(stut,Loci=="Cpb135")
stut_cpb135

## Loci N NExpBlanks ObsBlanks f.ExpBlanks.
## 8 Cpb135 72 5.432404 1 0.07545006

with(stut_cpb135,binom.test(ObsBlanks, N, f.ExpBlanks., alternative="less"))

##
## Exact binomial test
##
## data: ObsBlanks and N
## number of successes = 1, number of trials = 72, p-value = 0.02423
## alternative hypothesis: true probability of success is less than 0.07545006
## 95 percent confidence interval:
## 0.00000000 0.06419854
## sample estimates:
## probability of success
## 0.01388889

stut_cpb160<-subset(stut,Loci=="Cpb160")
stut_cpb160

## Loci N NExpBlanks ObsBlanks f.ExpBlanks.
## 9 Cpb160 72 2.247974 1 0.03122187

with(stut_cpb160,binom.test(ObsBlanks, N, f.ExpBlanks., alternative="less"))

##
## Exact binomial test
##
## data: ObsBlanks and N
## number of successes = 1, number of trials = 72, p-value = 0.3383
## alternative hypothesis: true probability of success is less than 0.03122187
## 95 percent confidence interval:
## 0.00000000 0.06419854
## sample estimates:
## probability of success
## 0.01388889

stut_cpb139<-subset(stut,Loci=="Cpb139")
stut_cpb139

## Loci N NExpBlanks ObsBlanks f.ExpBlanks.
## 10 Cpb139 72 0.08255574 1 0.001146608

with(stut_cpb139,binom.test(ObsBlanks, N, f.ExpBlanks., alternative="less"))

##
## Exact binomial test
##
## data: ObsBlanks and N
## number of successes = 1, number of trials = 72, p-value = 0.9968
## alternative hypothesis: true probability of success is less than 0.001146608
## 95 percent confidence interval:
## 0.00000000 0.06419854
## sample estimates:
## probability of success
## 0.01388889

stut_cpb190<-subset(stut,Loci=="Cpb190")
stut_cpb190

## Loci N NExpBlanks ObsBlanks f.ExpBlanks.
## 11 Cpb190 72 1.418029 1 0.01969485

with(stut_cpb190,binom.test(ObsBlanks, N, f.ExpBlanks., alternative="less"))

##
## Exact binomial test
##
## data: ObsBlanks and N
## number of successes = 1, number of trials = 72, p-value = 0.5842
## alternative hypothesis: true probability of success is less than 0.01969485
## 95 percent confidence interval:
## 0.00000000 0.06419854
## sample estimates:
## probability of success
## 0.01388889

# adjusted P value
p.adjust(c(0.3566,0.7657,0.9981,0.9985,0.9454,0.9968,1,0.02423,0.3383,0.9968,0.5842), method="BH")

## [1] 1.00000 1.00000 1.00000 1.00000 1.00000 1.00000 1.00000 0.26653 1.00000
## [10] 1.00000 1.00000

## Analysis the correlation between GST and Hs for CPB with 7 loci

#analysis the correlation between GST and HS for CPB with 7 loci

GstHs7 <- read.csv("Gst_Hs_7loci.csv",sep=",", row.names=NULL)
GstHs7

## Loci GST HS
## 1 Cpb54 0.02249719 0.869
## 2 Cpb62 0.01038062 0.858
## 3 Cpb84 0.01562500 0.882
## 4 Cpb122 0.03534778 0.846
## 5 Cpb112 0.02007084 0.830
## 6 Cpb160 0.03841676 0.826
## 7 Cpb190 0.07176471 0.789

with(GstHs7, cor.test(GST, HS, alternative="less", method="spearman"))

##
## Spearman's rank correlation rho
##
## data: GST and HS
## S = 98, p-value = 0.03313
## alternative hypothesis: true rho is less than 0
## sample estimates:
## rho
## -0.75

## Analysis the correlation between FIS and FST and number of blanks for CPB with 7 loci

#correlation between FIS and FST for CPB with 7 loci

fisfst7 <- read.csv("cpb_7loci_fis_fst.csv",sep=",", row.names=NULL)
fisfst7

## Loci FIS FST
## 1 Cpb54 -0.118 0.032
## 2 Cpb62 -0.116 0.015
## 3 Cpb84 0.087 0.023
## 4 Cpb122 -0.002 0.048
## 5 Cpb112 0.063 0.029
## 6 Cpb160 0.335 0.056
## 7 Cpb190 0.285 0.105

with(fisfst7, cor.test(FIS, FST, alternative="greater", method="spearman"))

##
## Spearman's rank correlation rho
##
## data: FIS and FST
## S = 26, p-value = 0.1179
## alternative hypothesis: true rho is greater than 0
## sample estimates:
## rho
## 0.5357143

#correlation between FIS and number of blanks with 5 loci
fisblank5 <- read.csv("Fis_blanks_5loci.csv",sep=",", row.names=NULL)
fisblank5

## Loci Blanks FIS
## 1 Cpb54 0 -0.118
## 2 Cpb84 0 0.087
## 3 Cpb112 0 0.063
## 4 Cpb160 1 0.335
## 5 Cpb190 1 0.285

with(fisblank5, cor.test(FIS, Blanks, alternative="greater", method="spearman"))

## Warning in cor.test.default(FIS, Blanks, alternative = "greater", method =
## "spearman"): Cannot compute exact p-value with ties

##
## Spearman's rank correlation rho
##
## data: FIS and Blanks
## S = 2.6795, p-value = 0.02883
## alternative hypothesis: true rho is greater than 0
## sample estimates:
## rho
## 0.8660254

## Analysis the Geographical discatnce between location for CPB with 7 loci

library(geosphere)
Longlat11<-read.table("Longlat11.txt", header = TRUE)
Longlat22<-read.table("Longlat22.txt", header = TRUE)
tabdistgeo<-data.frame(distGeo(Longlat11, Longlat22))
write.table(tabdistgeo,"tabdistgeo.txt",col=NA,sep="\t",dec=".")
dgeo <- read.table("tabdistgeo.txt",header = TRUE)
dgeo

## X distGeo.Longlat11..Longlat22.
## 1 1 2692605
## 2 2 2343372
## 3 3 1158721
## 4 4 2692605
## 5 5 2343372
## 6 6 1158721
## 7 7 2692605
## 8 8 2343372
## 9 9 1158721

## Analysis adjusted p value for LFB for LD values and for observed heterogeneity

p.adjust(c(00.8517, 0.5328, 0.3333, 0.6438, 0.7802, 0.7005, 0.9416, 0.5651, 0.4517, 0.8289, 0.0069, 1, 0.8056, 0.691, 0.6992, 1, 0.0503, 0.3089, 0.6856, 1, 0.6093, 0.7343, 1, 0.7965, 0.1931, 1, 0.8441, 0.7123, 1, 0.81, 1, 1, 0.574, 0.5147, 1, 1, 0.1716, 1, 0.4394, 0.5623, 1, 0.5261, 1, 0.5381, 0.6493, 0.8413, 1, 1, 1, 0.5727, 0.9562, 0.4851, 0.7081, 0.8287, 1), method="BY")

## [1] 1 1 1 1 1 1 1 1 1 1 1 1 1 1 1 1 1 1 1 1 1 1 1 1 1 1 1 1 1 1 1 1 1 1 1 1 1 1
## [39] 1 1 1 1 1 1 1 1 1 1 1 1 1 1 1 1 1

# adjusted P value for observed heterogeneity
p.adjust(c(0.7664, 0.2309, 0.2371, 1, 0.1652, 0.6948, 0.1608, 1, 0.01838, 0.9332, 0.07359), method="BH")

## [1] 1.0000000 0.4346833 0.4346833 1.0000000 0.4346833 1.0000000 0.4346833
## [8] 1.0000000 0.2021800 1.0000000 0.4047450

## Analysis for short allel dominance (SAD) for LFB with 11 loci

LFB_SAD <- read.csv("LFB_SAD.csv",sep=",", row.names=NULL)
LFB_SAD

## loci Allele Smallf p weight
## 1 Cpb14 234 -0.027 0.048 0.045696
## 2 Cpb14 236 -0.178 0.833 0.139111
## 3 Cpb14 242 -0.054 0.071 0.065959
## 4 Cpb14 245 -0.027 0.048 0.045696
## 5 Cpb55 254 -0.002 0.025 0.024375
## 6 Cpb55 256 -0.002 0.025 0.024375
## 7 Cpb55 258 1.000 0.050 0.047500
## 8 Cpb55 260 0.158 0.825 0.144375
## 9 Cpb55 262 -0.002 0.025 0.024375
## 10 Cpb55 265 -0.002 0.025 0.024375
## 11 Cpb55 271 -0.002 0.025 0.024375
## 12 Cpb54 169 -0.001 0.024 0.023424
## 13 Cpb54 173 -0.001 0.024 0.023424
## 14 Cpb54 231 -0.144 0.143 0.122551
## 15 Cpb54 249 -0.027 0.048 0.045696
## 16 Cpb54 253 -0.027 0.048 0.045696
## 17 Cpb54 255 -0.054 0.071 0.065959
## 18 Cpb54 257 1.000 0.048 0.045696
## 19 Cpb54 259 -0.227 0.548 0.247696
## 20 Cpb54 265 -0.001 0.024 0.023424
## 21 Cpb54 271 -0.001 0.024 0.023424
## 22 Cpb62 191 -0.001 0.024 0.023424
## 23 Cpb62 211 -0.001 0.976 0.023424
## 24 Cpb84 199 -0.001 0.024 0.023424
## 25 Cpb84 205 -0.027 0.048 0.045696
## 26 Cpb84 207 0.235 0.238 0.181356
## 27 Cpb84 209 0.321 0.286 0.204204
## 28 Cpb84 211 0.097 0.190 0.153900
## 29 Cpb84 213 -0.082 0.095 0.085975
## 30 Cpb84 215 -0.027 0.048 0.045696
## 31 Cpb84 219 -0.001 0.024 0.023424
## 32 Cpb84 233 -0.027 0.048 0.045696
## 33 Cpb122 180 -0.002 0.025 0.024375
## 34 Cpb122 182 0.020 0.225 0.174375
## 35 Cpb122 184 -0.167 0.300 0.210000
## 36 Cpb122 186 0.146 0.275 0.199375
## 37 Cpb122 188 -0.029 0.050 0.047500
## 38 Cpb122 190 -0.088 0.100 0.090000
## 39 Cpb122 192 -0.002 0.025 0.024375
## 40 Cpb112 182 -0.119 0.125 0.109375
## 41 Cpb112 184 0.336 0.125 0.109375
## 42 Cpb112 186 -0.057 0.075 0.069375
## 43 Cpb112 190 0.463 0.100 0.090000
## 44 Cpb112 216 -0.057 0.075 0.069375
## 45 Cpb112 274 -0.002 0.025 0.024375
## 46 Cpb112 296 -0.002 0.025 0.024375
## 47 Cpb112 300 0.781 0.125 0.109375
## 48 Cpb112 302 -0.057 0.075 0.069375
## 49 Cpb112 304 0.395 0.200 0.160000
## 50 Cpb112 306 -0.002 0.025 0.024375
## 51 Cpb112 308 -0.002 0.025 0.024375
## 52 Cpb135 210 0.655 0.071 0.065959
## 53 Cpb135 216 0.244 0.857 0.122551
## 54 Cpb135 220 -0.027 0.048 0.045696
## 55 Cpb135 240 -0.001 0.024 0.023424
## 56 Cpb160 282 -0.057 0.075 0.069375
## 57 Cpb160 286 -0.002 0.025 0.024375
## 58 Cpb160 288 -0.029 0.050 0.047500
## 59 Cpb160 292 0.781 0.125 0.109375
## 60 Cpb160 294 0.121 0.475 0.249375
## 61 Cpb160 296 -0.002 0.025 0.024375
## 62 Cpb160 298 -0.029 0.050 0.047500
## 63 Cpb160 300 -0.119 0.125 0.109375
## 64 Cpb160 302 1.000 0.050 0.047500
## 65 Cpb139 216 -0.001 0.024 0.023424
## 66 Cpb139 219 0.655 0.071 0.065959
## 67 Cpb139 221 -0.001 0.024 0.023424
## 68 Cpb139 224 -0.251 0.214 0.168204
## 69 Cpb139 228 -0.251 0.214 0.168204
## 70 Cpb139 230 -0.054 0.071 0.065959
## 71 Cpb139 232 -0.027 0.048 0.045696
## 72 Cpb139 236 -0.144 0.143 0.122551
## 73 Cpb139 280 -0.001 0.024 0.023424
## 74 Cpb139 288 -0.001 0.024 0.023424
## 75 Cpb139 291 -0.001 0.024 0.023424
## 76 Cpb139 293 -0.054 0.071 0.065959
## 77 Cpb139 297 -0.001 0.024 0.023424
## 78 Cpb139 299 -0.001 0.024 0.023424
## 79 Cpb190 281 0.461 0.105 0.093975
## 80 Cpb190 285 -0.003 0.026 0.025324
## 81 Cpb190 289 1.000 0.053 0.050191
## 82 Cpb190 291 0.584 0.421 0.243759
## 83 Cpb190 293 0.294 0.237 0.180831
## 84 Cpb190 297 1.000 0.053 0.050191
## 85 Cpb190 299 -0.003 0.026 0.025324
## 86 Cpb190 305 -0.003 0.026 0.025324
## 87 Cpb190 307 -0.003 0.026 0.025324
## 88 Cpb190 323 -0.003 0.026 0.025324

LFB_SAD$weight<- as.numeric(LFB_SAD$weight)
LFB_SAD$Allele <- as.numeric(LFB_SAD$Allele)

LFB_SAD_Cpb14 <- subset(LFB_SAD,loci=="Cpb14")
LFB_SAD_Cpb14

## loci Allele Smallf p weight
## 1 Cpb14 234 -0.027 0.048 0.045696
## 2 Cpb14 236 -0.178 0.833 0.139111
## 3 Cpb14 242 -0.054 0.071 0.065959
## 4 Cpb14 245 -0.027 0.048 0.045696

lm_LFB_SAD_Cpb14 <- lm(Smallf ~ Allele, data=LFB_SAD_Cpb14, weights=weight)
summary(lm_LFB_SAD_Cpb14)

##
## Call:
## lm(formula = Smallf ~ Allele, data = LFB_SAD_Cpb14, weights = weight)
##
## Weighted Residuals:
## 1 2 3 4
## 0.025659 -0.018846 0.003798 0.002660
##
## Coefficients:
## Estimate Std. Error t value Pr(>|t|)
## (Intercept) -2.435734 2.548281 -0.956 0.440
## Allele 0.009781 0.010687 0.915 0.457
##
## Residual standard error: 0.02275 on 2 degrees of freedom
## Multiple R-squared: 0.2952, Adjusted R-squared: -0.05724
## F-statistic: 0.8376 on 1 and 2 DF, p-value: 0.4567

LFB_SAD_Cpb55 <- subset(LFB_SAD,loci=="Cpb55")
LFB_SAD_Cpb55

## loci Allele Smallf p weight
## 5 Cpb55 254 -0.002 0.025 0.024375
## 6 Cpb55 256 -0.002 0.025 0.024375
## 7 Cpb55 258 1.000 0.050 0.047500
## 8 Cpb55 260 0.158 0.825 0.144375
## 9 Cpb55 262 -0.002 0.025 0.024375
## 10 Cpb55 265 -0.002 0.025 0.024375
## 11 Cpb55 271 -0.002 0.025 0.024375

lm_LFB_SAD_Cpb55 <- lm(Smallf ~ Allele, data=LFB_SAD_Cpb55, weights=weight)
summary(lm_LFB_SAD_Cpb55)

##
## Call:
## lm(formula = Smallf ~ Allele, data = LFB_SAD_Cpb55, weights = weight)
##
## Weighted Residuals:
## 5 6 7 8 9 10 11
## -0.058031 -0.050798 0.157566 -0.027626 -0.029098 -0.018248 0.003452
##
## Coefficients:
## Estimate Std. Error t value Pr(>|t|)
## (Intercept) 6.25364 9.34400 0.669 0.533
## Allele -0.02317 0.03589 -0.645 0.547
##
## Residual standard error: 0.08091 on 5 degrees of freedom
## Multiple R-squared: 0.07691, Adjusted R-squared: -0.1077
## F-statistic: 0.4166 on 1 and 5 DF, p-value: 0.5471

LFB_SAD_Cpb54 <- subset(LFB_SAD,loci=="Cpb54")
LFB_SAD_Cpb54

## loci Allele Smallf p weight
## 12 Cpb54 169 -0.001 0.024 0.023424
## 13 Cpb54 173 -0.001 0.024 0.023424
## 14 Cpb54 231 -0.144 0.143 0.122551
## 15 Cpb54 249 -0.027 0.048 0.045696
## 16 Cpb54 253 -0.027 0.048 0.045696
## 17 Cpb54 255 -0.054 0.071 0.065959
## 18 Cpb54 257 1.000 0.048 0.045696
## 19 Cpb54 259 -0.227 0.548 0.247696
## 20 Cpb54 265 -0.001 0.024 0.023424
## 21 Cpb54 271 -0.001 0.024 0.023424

lm_LFB_SAD_Cpb54 <- lm(Smallf ~ Allele, data=LFB_SAD_Cpb54, weights=weight)
summary(lm_LFB_SAD_Cpb54)

##
## Call:
## lm(formula = Smallf ~ Allele, data = LFB_SAD_Cpb54, weights = weight)
##
## Weighted Residuals:
## Min 1Q Median 3Q Max
## -0.087676 0.000717 0.006368 0.008091 0.224654
##
## Coefficients:
## Estimate Std. Error t value Pr(>|t|)
## (Intercept) -6.328e-02 1.112e+00 -0.057 0.956
## Allele 4.805e-05 4.487e-03 0.011 0.992
##
## Residual standard error: 0.08624 on 8 degrees of freedom
## Multiple R-squared: 1.434e-05, Adjusted R-squared: -0.125
## F-statistic: 0.0001147 on 1 and 8 DF, p-value: 0.9917

LFB_SAD_Cpb62 <- subset(LFB_SAD,loci=="Cpb62")
LFB_SAD_Cpb62

## loci Allele Smallf p weight
## 22 Cpb62 191 -0.001 0.024 0.023424
## 23 Cpb62 211 -0.001 0.976 0.023424

lm_LFB_SAD_Cpb62 <- lm(Smallf ~ Allele, data=LFB_SAD_Cpb62, weights=weight)
summary(lm_LFB_SAD_Cpb62)

##
## Call:
## lm(formula = Smallf ~ Allele, data = LFB_SAD_Cpb62, weights = weight)
##
## Residuals:
## ALL 2 residuals are 0: no residual degrees of freedom!
##
## Coefficients:
## Estimate Std. Error t value Pr(>|t|)
## (Intercept) -0.001 NaN NaN NaN
## Allele 0.000 NaN NaN NaN
##
## Residual standard error: NaN on 0 degrees of freedom
## Multiple R-squared: NaN, Adjusted R-squared: NaN
## F-statistic: NaN on 1 and 0 DF, p-value: NA

LFB_SAD_Cpb84 <- subset(LFB_SAD,loci=="Cpb84")
LFB_SAD_Cpb84

## loci Allele Smallf p weight
## 24 Cpb84 199 -0.001 0.024 0.023424
## 25 Cpb84 205 -0.027 0.048 0.045696
## 26 Cpb84 207 0.235 0.238 0.181356
## 27 Cpb84 209 0.321 0.286 0.204204
## 28 Cpb84 211 0.097 0.190 0.153900
## 29 Cpb84 213 -0.082 0.095 0.085975
## 30 Cpb84 215 -0.027 0.048 0.045696
## 31 Cpb84 219 -0.001 0.024 0.023424
## 32 Cpb84 233 -0.027 0.048 0.045696

lm_LFB_SAD_Cpb84 <- lm(Smallf ~ Allele, data=LFB_SAD_Cpb84, weights=weight)
summary(lm_LFB_SAD_Cpb84)

##
## Call:
## lm(formula = Smallf ~ Allele, data = LFB_SAD_Cpb84, weights = weight)
##
## Weighted Residuals:
## Min 1Q Median 3Q Max
## -0.05860 -0.03876 -0.01572 0.01007 0.07444
##
## Coefficients:
## Estimate Std. Error t value Pr(>|t|)
## (Intercept) 2.162247 1.662400 1.301 0.235
## Allele -0.009598 0.007882 -1.218 0.263
##
## Residual standard error: 0.04555 on 7 degrees of freedom
## Multiple R-squared: 0.1748, Adjusted R-squared: 0.05694
## F-statistic: 1.483 on 1 and 7 DF, p-value: 0.2628

LFB_SAD_Cpb122 <- subset(LFB_SAD,loci=="Cpb122")
LFB_SAD_Cpb122

## loci Allele Smallf p weight
## 33 Cpb122 180 -0.002 0.025 0.024375
## 34 Cpb122 182 0.020 0.225 0.174375
## 35 Cpb122 184 -0.167 0.300 0.210000
## 36 Cpb122 186 0.146 0.275 0.199375
## 37 Cpb122 188 -0.029 0.050 0.047500
## 38 Cpb122 190 -0.088 0.100 0.090000
## 39 Cpb122 192 -0.002 0.025 0.024375

lm_LFB_SAD_Cpb122 <- lm(Smallf ~ Allele, data=LFB_SAD_Cpb122, weights=weight)
summary(lm_LFB_SAD_Cpb122)

##
## Call:
## lm(formula = Smallf ~ Allele, data = LFB_SAD_Cpb122, weights = weight)
##
## Weighted Residuals:
## 33 34 35 36 37 38 39
## 0.0035314 0.0171364 -0.0685301 0.0713856 -0.0040776 -0.0243874 0.0001759
##
## Coefficients:
## Estimate Std. Error t value Pr(>|t|)
## (Intercept) -0.347007 3.375568 -0.103 0.922
## Allele 0.001791 0.018230 0.098 0.926
##
## Residual standard error: 0.04628 on 5 degrees of freedom
## Multiple R-squared: 0.001927, Adjusted R-squared: -0.1977
## F-statistic: 0.009652 on 1 and 5 DF, p-value: 0.9256

LFB_SAD_Cpb112 <- subset(LFB_SAD,loci=="Cpb112")
LFB_SAD_Cpb112

## loci Allele Smallf p weight
## 40 Cpb112 182 -0.119 0.125 0.109375
## 41 Cpb112 184 0.336 0.125 0.109375
## 42 Cpb112 186 -0.057 0.075 0.069375
## 43 Cpb112 190 0.463 0.100 0.090000
## 44 Cpb112 216 -0.057 0.075 0.069375
## 45 Cpb112 274 -0.002 0.025 0.024375
## 46 Cpb112 296 -0.002 0.025 0.024375
## 47 Cpb112 300 0.781 0.125 0.109375
## 48 Cpb112 302 -0.057 0.075 0.069375
## 49 Cpb112 304 0.395 0.200 0.160000
## 50 Cpb112 306 -0.002 0.025 0.024375
## 51 Cpb112 308 -0.002 0.025 0.024375

lm_LFB_SAD_Cpb112 <- lm(Smallf ~ Allele, data=LFB_SAD_Cpb112, weights=weight)
summary(lm_LFB_SAD_Cpb112)

##
## Call:
## lm(formula = Smallf ~ Allele, data = LFB_SAD_Cpb112, weights = weight)
##
## Weighted Residuals:
## Min 1Q Median 3Q Max
## -0.10152 -0.05542 -0.04909 0.03657 0.15083
##
## Coefficients:
## Estimate Std. Error t value Pr(>|t|)
## (Intercept) -0.198331 0.399698 -0.496 0.630
## Allele 0.001744 0.001592 1.096 0.299
##
## Residual standard error: 0.08419 on 10 degrees of freedom
## Multiple R-squared: 0.1072, Adjusted R-squared: 0.01793
## F-statistic: 1.201 on 1 and 10 DF, p-value: 0.2988

LFB_SAD_Cpb135 <- subset(LFB_SAD,loci=="Cpb135")
LFB_SAD_Cpb135

## loci Allele Smallf p weight
## 52 Cpb135 210 0.655 0.071 0.065959
## 53 Cpb135 216 0.244 0.857 0.122551
## 54 Cpb135 220 -0.027 0.048 0.045696
## 55 Cpb135 240 -0.001 0.024 0.023424

lm_LFB_SAD_Cpb135 <- lm(Smallf ~ Allele, data=LFB_SAD_Cpb135, weights=weight)
summary(lm_LFB_SAD_Cpb135)

##
## Call:
## lm(formula = Smallf ~ Allele, data = LFB_SAD_Cpb135, weights = weight)
##
## Weighted Residuals:
## 52 53 54 55
## 0.05417 -0.02287 -0.05269 0.03499
##
## Coefficients:
## Estimate Std. Error t value Pr(>|t|)
## (Intercept) 5.15996 3.30774 1.560 0.259
## Allele -0.02246 0.01521 -1.477 0.278
##
## Residual standard error: 0.06107 on 2 degrees of freedom
## Multiple R-squared: 0.5216, Adjusted R-squared: 0.2824
## F-statistic: 2.18 on 1 and 2 DF, p-value: 0.2778

LFB_SAD_Cpb160 <- subset(LFB_SAD,loci=="Cpb160")
LFB_SAD_Cpb160

## loci Allele Smallf p weight
## 56 Cpb160 282 -0.057 0.075 0.069375
## 57 Cpb160 286 -0.002 0.025 0.024375
## 58 Cpb160 288 -0.029 0.050 0.047500
## 59 Cpb160 292 0.781 0.125 0.109375
## 60 Cpb160 294 0.121 0.475 0.249375
## 61 Cpb160 296 -0.002 0.025 0.024375
## 62 Cpb160 298 -0.029 0.050 0.047500
## 63 Cpb160 300 -0.119 0.125 0.109375
## 64 Cpb160 302 1.000 0.050 0.047500

lm_LFB_SAD_Cpb160 <- lm(Smallf ~ Allele, data=LFB_SAD_Cpb160, weights=weight)
summary(lm_LFB_SAD_Cpb160)

##
## Call:
## lm(formula = Smallf ~ Allele, data = LFB_SAD_Cpb160, weights = weight)
##
## Weighted Residuals:
## Min 1Q Median 3Q Max
## -0.12710 -0.03965 -0.03498 -0.01809 0.19917
##
## Coefficients:
## Estimate Std. Error t value Pr(>|t|)
## (Intercept) -2.97978 7.15346 -0.417 0.689
## Allele 0.01082 0.02436 0.444 0.670
##
## Residual standard error: 0.1128 on 7 degrees of freedom
## Multiple R-squared: 0.0274, Adjusted R-squared: -0.1115
## F-statistic: 0.1972 on 1 and 7 DF, p-value: 0.6704

LFB_SAD_Cpb139 <- subset(LFB_SAD,loci=="Cpb139")
LFB_SAD_Cpb139

## loci Allele Smallf p weight
## 65 Cpb139 216 -0.001 0.024 0.023424
## 66 Cpb139 219 0.655 0.071 0.065959
## 67 Cpb139 221 -0.001 0.024 0.023424
## 68 Cpb139 224 -0.251 0.214 0.168204
## 69 Cpb139 228 -0.251 0.214 0.168204
## 70 Cpb139 230 -0.054 0.071 0.065959
## 71 Cpb139 232 -0.027 0.048 0.045696
## 72 Cpb139 236 -0.144 0.143 0.122551
## 73 Cpb139 280 -0.001 0.024 0.023424
## 74 Cpb139 288 -0.001 0.024 0.023424
## 75 Cpb139 291 -0.001 0.024 0.023424
## 76 Cpb139 293 -0.054 0.071 0.065959
## 77 Cpb139 297 -0.001 0.024 0.023424
## 78 Cpb139 299 -0.001 0.024 0.023424

lm_LFB_SAD_Cpb139 <- lm(Smallf ~ Allele, data=LFB_SAD_Cpb139, weights=weight)
summary(lm_LFB_SAD_Cpb139)

##
## Call:
## lm(formula = Smallf ~ Allele, data = LFB_SAD_Cpb139, weights = weight)
##
## Weighted Residuals:
## Min 1Q Median 3Q Max
## -0.068792 0.002179 0.008328 0.011059 0.190581
##
## Coefficients:
## Estimate Std. Error t value Pr(>|t|)
## (Intercept) -0.179487 0.607977 -0.295 0.773
## Allele 0.000422 0.002507 0.168 0.869
##
## Residual standard error: 0.06265 on 12 degrees of freedom
## Multiple R-squared: 0.002355, Adjusted R-squared: -0.08078
## F-statistic: 0.02833 on 1 and 12 DF, p-value: 0.8691

LFB_SAD_Cpb190 <- subset(LFB_SAD,loci=="Cpb190")
LFB_SAD_Cpb190

## loci Allele Smallf p weight
## 79 Cpb190 281 0.461 0.105 0.093975
## 80 Cpb190 285 -0.003 0.026 0.025324
## 81 Cpb190 289 1.000 0.053 0.050191
## 82 Cpb190 291 0.584 0.421 0.243759
## 83 Cpb190 293 0.294 0.237 0.180831
## 84 Cpb190 297 1.000 0.053 0.050191
## 85 Cpb190 299 -0.003 0.026 0.025324
## 86 Cpb190 305 -0.003 0.026 0.025324
## 87 Cpb190 307 -0.003 0.026 0.025324
## 88 Cpb190 323 -0.003 0.026 0.025324

lm_LFB_SAD_Cpb190 <- lm(Smallf ~ Allele, data=LFB_SAD_Cpb190, weights=weight)
summary(lm_LFB_SAD_Cpb190)

##
## Call:
## lm(formula = Smallf ~ Allele, data = LFB_SAD_Cpb190, weights = weight)
##
## Weighted Residuals:
## Min 1Q Median 3Q Max
## -0.08980 -0.05603 -0.04324 0.03797 0.13574
##
## Coefficients:
## Estimate Std. Error t value Pr(>|t|)
## (Intercept) 4.53181 3.44762 1.314 0.225
## Allele -0.01393 0.01178 -1.183 0.271
##
## Residual standard error: 0.08324 on 8 degrees of freedom
## Multiple R-squared: 0.1489, Adjusted R-squared: 0.04253
## F-statistic: 1.4 on 1 and 8 DF, p-value: 0.2707

## Analysis the correlation of FIS and number of blanks for LFB with 11 loci

# correlation of FIS and number of blanks for LFB with 11 loci

lfbfisblank11 <- read.csv("LFB_11loci_fis_blank.csv",sep=",", row.names=NULL)
lfbfisblank11

## loci Blanks FIS
## 1 Cpb14 0 -0.103
## 2 Cpb55 1 0.226
## 3 Cpb54 0 -0.050
## 4 Cpb62 0 -0.001
## 5 Cpb84 0 0.139
## 6 Cpb122 1 -0.015
## 7 Cpb112 1 0.231
## 8 Cpb135 0 0.281
## 9 Cpb160 1 0.200
## 10 Cpb139 0 -0.076
## 11 Cpb190 2 0.456

with(lfbfisblank11, cor.test(FIS, Blanks, alternative="greater", method="spearman"))

## Warning in cor.test.default(FIS, Blanks, alternative = "greater", method =
## "spearman"): Cannot compute exact p-value with ties

##
## Spearman's rank correlation rho
##
## data: FIS and Blanks
## S = 91.059, p-value = 0.02905
## alternative hypothesis: true rho is greater than 0
## sample estimates:
## rho
## 0.5860943

## Analysis of male female divergence for supplementary file 2

#male female divergence_supplementary file 2
subfile2 <- read.csv("malefemale_divergence.csv",sep=",", row.names=NULL)
subfile2

## loci FIS.Females FIS.Males
## 1 Cpb14 NA 0.001
## 2 Cpb14 -0.032 -0.001
## 3 Cpb14 0.207 0.486
## 4 Cpb14 0.001 -0.099
## 5 Cpb14 0.204 -0.075
## 6 Cpb14 0.001 1.000
## 7 Cpb14 0.154 0.396
## 8 Cpb14 1.000 NA
## 9 Cpb14 0.001 0.001
## 10 Cpb14 NA 1.000
## 11 Cpb14 0.001 0.001
## 12 Cpb14 1.000 NA
## 13 Cpb14 0.001 NA
## 14 Cpb14 0.214 0.333
## 15 Cpb112 NA -0.253
## 16 Cpb112 -0.007 0.271
## 17 Cpb112 -0.007 -0.056
## 18 Cpb112 0.413 NA
## 19 Cpb112 0.002 0.001
## 20 Cpb112 0.002 0.001
## 21 Cpb112 NA 0.001
## 22 Cpb112 0.002 0.001
## 23 Cpb112 0.002 NA
## 24 Cpb112 -0.042 -0.003
## 25 Cpb112 NA 0.001
## 26 Cpb112 NA -0.003
## 27 Cpb112 -0.312 -0.087
## 28 Cpb112 0.002 0.001
## 29 Cpb112 0.332 0.139
## 30 Cpb112 0.383 0.148
## 31 Cpb112 0.187 -0.050
## 32 Cpb112 -0.098 -0.031
## 33 Cpb112 -0.024 0.001
## 34 Cpb112 0.002 NA
## 35 Cpb122 NA 0.005
## 36 Cpb122 -0.003 -0.196
## 37 Cpb122 -0.003 -0.181
## 38 Cpb122 -0.229 0.514
## 39 Cpb122 -0.038 0.673
## 40 Cpb122 -0.113 0.010
## 41 Cpb122 -0.032 -0.043
## 42 Cpb122 -0.030 -0.019
## 43 Cpb122 -0.111 0.208
## 44 Cpb122 -0.346 -0.209
## 45 Cpb122 0.000 0.351
## 46 Cpb122 -0.057 0.234
## 47 Cpb122 0.129 -0.240
## 48 Cpb122 0.001 -0.086
## 49 Cpb122 0.001 NA
## 50 Cpb122 -0.072 NA
## 51 Cpb122 0.001 0.014
## 52 Cpb122 NA -0.034
## 53 Cpb122 0.001 0.014
## 54 Cpb122 NA 0.010

subfile2_Cpb14 <- subset(subfile2,loci=="Cpb14")
subfile2_Cpb14

## loci FIS.Females FIS.Males
## 1 Cpb14 NA 0.001
## 2 Cpb14 -0.032 -0.001
## 3 Cpb14 0.207 0.486
## 4 Cpb14 0.001 -0.099
## 5 Cpb14 0.204 -0.075
## 6 Cpb14 0.001 1.000
## 7 Cpb14 0.154 0.396
## 8 Cpb14 1.000 NA
## 9 Cpb14 0.001 0.001
## 10 Cpb14 NA 1.000
## 11 Cpb14 0.001 0.001
## 12 Cpb14 1.000 NA
## 13 Cpb14 0.001 NA
## 14 Cpb14 0.214 0.333

with(subfile2_Cpb14, wilcox.test(FIS.Females, FIS.Males, alternative='two.sided', paired=TRUE))

## Warning in wilcox.test.default(FIS.Females, FIS.Males, alternative =
## "two.sided", : cannot compute exact p-value with zeroes

##
## Wilcoxon signed rank test with continuity correction
##
## data: FIS.Females and FIS.Males
## V = 7, p-value = 0.2719
## alternative hypothesis: true location shift is not equal to 0

subfile2_Cpb112 <- subset(subfile2,loci=="Cpb112")
subfile2_Cpb112

## loci FIS.Females FIS.Males
## 15 Cpb112 NA -0.253
## 16 Cpb112 -0.007 0.271
## 17 Cpb112 -0.007 -0.056
## 18 Cpb112 0.413 NA
## 19 Cpb112 0.002 0.001
## 20 Cpb112 0.002 0.001
## 21 Cpb112 NA 0.001
## 22 Cpb112 0.002 0.001
## 23 Cpb112 0.002 NA
## 24 Cpb112 -0.042 -0.003
## 25 Cpb112 NA 0.001
## 26 Cpb112 NA -0.003
## 27 Cpb112 -0.312 -0.087
## 28 Cpb112 0.002 0.001
## 29 Cpb112 0.332 0.139
## 30 Cpb112 0.383 0.148
## 31 Cpb112 0.187 -0.050
## 32 Cpb112 -0.098 -0.031
## 33 Cpb112 -0.024 0.001
## 34 Cpb112 0.002 NA

with(subfile2_Cpb112, wilcox.test(FIS.Females, FIS.Males, alternative='two.sided', paired=TRUE))

## Warning in wilcox.test.default(FIS.Females, FIS.Males, alternative =
## "two.sided", : cannot compute exact p-value with ties

##
## Wilcoxon signed rank test with continuity correction
##
## data: FIS.Females and FIS.Males
## V = 49, p-value = 0.8334
## alternative hypothesis: true location shift is not equal to 0

# Bh adjusted P value for supplementary file 2
p.adjust(c(0.6874, 0.6874, 0.3165, 0.6874, 0.2241, 0.0151, 0.0684, 0.6874, 0.179, 0.6874, 0.2808), method="BH")

## [1] 0.68740 0.68740 0.58025 0.68740 0.58025 0.16610 0.37620 0.68740 0.58025
## [10] 0.68740 0.58025

subfile2_Cpb122 <- subset(subfile2,loci=="Cpb122")
subfile2_Cpb122

## loci FIS.Females FIS.Males
## 35 Cpb122 NA 0.005
## 36 Cpb122 -0.003 -0.196
## 37 Cpb122 -0.003 -0.181
## 38 Cpb122 -0.229 0.514
## 39 Cpb122 -0.038 0.673
## 40 Cpb122 -0.113 0.010
## 41 Cpb122 -0.032 -0.043
## 42 Cpb122 -0.030 -0.019
## 43 Cpb122 -0.111 0.208
## 44 Cpb122 -0.346 -0.209
## 45 Cpb122 0.000 0.351
## 46 Cpb122 -0.057 0.234
## 47 Cpb122 0.129 -0.240
## 48 Cpb122 0.001 -0.086
## 49 Cpb122 0.001 NA
## 50 Cpb122 -0.072 NA
## 51 Cpb122 0.001 0.014
## 52 Cpb122 NA -0.034
## 53 Cpb122 0.001 0.014
## 54 Cpb122 NA 0.010

with(subfile2_Cpb122, wilcox.test(FIS.Females, FIS.Males, alternative='two.sided', paired=TRUE))

## Warning in wilcox.test.default(FIS.Females, FIS.Males, alternative =
## "two.sided", : cannot compute exact p-value with ties

##
## Wilcoxon signed rank test with continuity correction
##
## data: FIS.Females and FIS.Males
## V = 36, p-value = 0.1819
## alternative hypothesis: true location shift is not equal to 0

## 
