# Supplementary material for: Development and characterization of microsatellite markers for population genetics of the cocoa pod borer Conopomorpha cramerella (Snellen) (Lepidoptera: Gracillaridae)
Source: PLoS One. 2024 Apr 11;19(4):e0297662. doi: 10.1371/journal.pone.0297662 (PMC11008836; doi:10.1371/journal.pone.0297662)
Supplement: S2 File — (PDF) [file pone.0297662.s011.pdf]

Inclusivity in global research

PLOS' policy on inclusivity in global research aims to improve transparency in the reporting of research performed outside of researchers' own country or community and ensures that PLOS publications reporting global research adhere to high standards for research ethics and authorship. Authors of relevant research articles may be asked to complete the questionnaire below, which outlines ethical, cultural, and scientific considerations specific to inclusivity in global research. This questionnaire may be requested when researchers have travelled to a different country to conduct research, if research uses samples collected in another country, research with Indigenous populations or their lands, or if research is on cultural artefacts. Researchers travelling to another country solely to use laboratory equipment will not normally be required to complete the questionnaire. However, the questionnaire can be requested at the journal's discretion for any submission – if you have been requested to complete this questionnaire by the PLOS journal you submitted to, please do so.

Please complete the questionnaire below and include this as a Supporting Information file with your manuscript. Note that if your paper is accepted for publication, this checklist will be published with your article in the supporting information files. Please ensure that you reference the checklist in the main body of your manuscript. We suggest adding a subsection 'Inclusivity in global research' to your Methods section and adding the following sentence: "Additional information regarding the ethical, cultural, and scientific considerations specific to inclusivity in global research is included in the Supporting Information (SX Checklist)"

The questions have been designed to be applicable to a wide range of study types, and there are subsections for both human subjects research and non-human subjects research. If any of the questions are not relevant to your research please mark them as "N/A" as appropriate.

Ethical considerations, permits and authorship

This section is applicable to all research types.

Provide details as to who granted permissions and/or consent for the study to take place in the Methods section of your manuscript. This should include the names of **all** ethics boards, governmental organizations, community leaders or other bodies that provided approval for the study. If individuals provided approval refer to these people by their role or title but do not list their name(s).

Reported on page number:
The research work reported in this manuscript were conducted in the framework of the activities of the Regional Technical Cooperation project RAS5086 funded by the International Atomic Egengy Agency and agreed by the participating Institutions including the ones co-authoring this study:

1- Biological Control Research Unit, Center for Natural Science and Environmental Research, De La Salle University, Manila, Philippines
2- Centre for Cocoa Biotechnology Research, Malaysian Cocoa Board, Sabah, Malaysia
3- Intertryp, Univ Montpellier, Cirad, IRD, Montpellier, France
4- IRD, UMR Intertryp, Cirad, Campus International de Baillarguet, 34398 Montpellier Cedex 5, France
5- Lembaga Koko Malaysia, Bagan Datuk, Perak, Malaysia
6- Plant Protection Research Institute, Duc Thang, Hanoi, Vietnam.
7- Indonesian Coffee and Cocoa Research Institute, Jember, Indonesia
8- Mars Cocoa Research Centre, Mars Wrigley, Sulawesi Selatan, Indonesia
9- Mars Wrigley, Centre for Tropical Environmental & Sustainability Science, James Cook University Nguma-bada Campus, Smithfield, Australia
10- Insect Pest Control Laboratory, Joint FAO/IAEA Centre of Nuclear Techniques in Food and Agriculture, Vienna, Austria

If there were any deviations from the study protocol after approval was obtained please provide details of these changes in the Methods section of your manuscript.

Reported on page number: No deviations occurred

Did this study involve local collaborators that are residents of the country where the research was conducted or members of the community studied? If you do not have any authors from said communities, please provide an explanation for this below.

All local collaborators co-author this manuscript

Everyone listed as an author should meet PLOS' criteria for authorship and all individuals who meet these criteria should be included in the author byline, rather than the acknowledgements. For further information please see the journal's Authorship Policy. **Error! Hyperlink reference not valid.**

Human subjects research (e.g. health research, medical research, cross-cultural psychology)

Did you obtain written informed consent from a representative of the local community or region before the research took place? How did you establish who speaks for the community? Details of written informed consent obtained from study participants should be reported separately in the Methods section of your manuscript.

N/A

How did members of the local community provide input on the aims of the research investigation, its methodology, and its anticipated outcome(s)?

N/A

When engaging with the local community, how did you ensure that the informed consent documents and other materials could be understood by local stakeholders?

N/A

N/A

Will the findings of the research be made available in an understandable format to stakeholders in the community where the study was conducted (e.g. via a presentation, summary report, copies of publications, etc.)? Please provide details of how this will be achieved.

Non-human subjects research using specimens/ animals collected as part of the study, or those housed in archival collections. Examples include archaeology, paleontology, botany and zoology.

Did the permission you obtained from a local authority to perform the study include an agreement on access to outputs and benefit sharing? This may include procedures to enable fair distribution of the benefits and resources arising from the research performed. Please include any details of Prior Informed Consent and Benefit Sharing Agreements obtained. These may be required by field-specific regulations, for example the Convention on Biological Diversity (CBD) and the associated Nagoya Protocol.

The research work reported in this manuscript is part of the activities conducted in the framework of the Regional Technical Cooperation project RAS5086 funded by the International Atomic Egengy Agency and agreed by the participating Institutions including the ones co-authoring this study:

1- Biological Control Research Unit, Center for Natural Science and Environmental Research, De La Salle University, Manila, Philippines
2- Centre for Cocoa Biotechnology Research, Malaysian Cocoa Board, Sabah, Malaysia
3- Intertryp, Univ Montpellier, Cirad, IRD, Montpellier, France
4- IRD, UMR Intertryp, Cirad, Campus International de Baillarguet, 34398 Montpellier Cedex 5, France
5- Lembaga Koko Malaysia, Bagan Datuk, Perak, Malaysia
6- Plant Protection Research Institute, Duc Thang, Hanoi, Vietnam.
7- Indonesian Coffee and Cocoa Research Institute, Jember, Indonesia
8- Mars Cocoa Research Centre, Mars Wrigley, Sulawesi Selatan, Indonesia
9- Mars Wrigley, Centre for Tropical Environmental & Sustainability Science, James Cook University Ngumabada Campus, Smithfield, Australia
10- Insect Pest Control Laboratory, Joint FAO/IAEA Centre of Nuclear Techniques in Food and Agriculture, Vienna, Austria

If the material used in your study was imported, please A) provide the year it was imported and B) indicate whether permits were obtained to import/export the materials used, C) provide details of any permits obtained. If this information is not available, please indicate this.

The samples (preserved in propylene-glycol) arrived at the Insect Pest Control Laboratory between 2019-2021 with all the necessary import permits (2023 0.259.064), see document below). Within the Regional Technical Cooperation project RAS5086 agreement, no export permit ws required in the countries where samples we collected.

If you used archival specimens, please state how the material used in your study was acquired by the institute it is held in and provide details of any permits obtained for the original excavations/ sample collection. If this information is not available, please indicate this.

No archival materials used in this study

How was the potential cultural significance of the materials collected in your study to local communities considered in your research design? Were Indigenous peoples and/or local researchers and institutions involved with archaeological excavations / collection of specimens? If so, please provide a description of their involvement.

N/A

If your manuscript includes photographs of human remains please indicate whether authors obtained permission from descendants or affiliated cultural communities to do so.

N/A

Bundesministerium
Soziales, Gesundheit, Pflege
und Konsumentenschutz

sozialministerium.at

BMSGPK-Gesundheit - III/B/16 (Tierärztliches Berufsrecht, Tiergesundheits- und Tierschutzrecht sowie weiter rechtliche Angelegenheiten im Veterinärwesen)

Mag. Georg Brandl
Sachbearbeiter

georg.brandl@gesundheitsministerium.gv.at
+43 1 711 00-644813
Postanschrift: Stubenring 1, 1010 Wien
Radetzkystraße 2, 1030 Wien

E-Mail-Antworten sind bitte unter Anführung der Geschäftszahl an post@sozialministerium.at zu richten.

Joint FAO/IAEA Division
Insect Pest Control
zH M. Vreysen
Wagramerstrasse 5
1040 Wien

M.Vreysen@iaea.org;S.Beckham@iaea.org;B.Mayer-Karolyi@iaea.org

Geschäftszahl: 2023-0.259.064

Joint FAO/IAEA Division - Veterinärbehördliche Einfuhrbewilligung für lebende Puppen von Tsetsefliegen für wissenschaftliche Zwecke aus allen Drittstaaten

B e s c h e i d

Der Bundesminister für Soziales, Gesundheit, Pflege und Konsumentenschutz bewilligt der Joint FAO/IAEA Division, Insect Pest Control, Wagramerstrasse 5, 1040 Wien auf Grund des Antrags vom 3. März 2023 gemäß §§ 4, 4a und 4b des Tierseuchengesetzes, RGBl.Nr. 177/1909, zuletzt geändert durch das Bundesgesetz BGBl. I Nr. 258/2021, in Verbindung mit §§ 13 und 14 der Veterinärbehördlichen Einfuhrverordnung 2022 (VEVO 2022), BGBl. II Nr. 480/2022, die **Einfuhr** auch partienweise von

lebenden Puppen von Tsetsefliegen (*Glossina*) **für wissenschaftliche Zwecke**

aus **allen Drittstaaten**

nach **Österreich**
an den Bestimmungsort**: 2444 Seibersdorf, Friedensstrasse 1, Insect Pest Control Laboratory der Joint FAO/IAEA Agriculture & Biotechnology Laboratories**

bis längstens **30. April 2024**

unter nachstehenden Bedingungen und Auflagen:

1. **Veterinärbehördliche Bedingungen für die Einfuhr**

a) Für jede Sendung ist der Grenztierärztin/dem Grenztierarzt eine **Bestätigung des Absenders** der Sendung vorzulegen, in der bestätigt wird, dass der Herkunftsbetrieb und der Ursprungsbetrieb nicht in einem Gebiet liegen, das aus tierseuchenrechtlichen Gründen gesperrt ist.

a) Teile der Sendung dürfen während der Beförderung aus den Fahrzeugen nicht herausfallen oder heraussickern.

b) Die Beförderung auf dem Gebiet der Union muss ohne Aus-, Um- und Zuladung erfolgen. Ist im Notfall eine Aus-, Um- oder Zuladung erforderlich, so hat hiervon, sofern dies unter zollamtlicher Überwachung erfolgt, das Zollamt, ansonsten das Verkehrsunternehmen (Beförderer) die örtlich zuständige Behörde (in Österreich Bezirksverwaltungsbehörde - Amtstierärztin/Amtstierarzt) zu verständigen. Die Behörde hat diesen Vorgang zu überwachen und hierüber einen entsprechenden Vermerk auf dem GGED (Gemeinsames Gesundheitseinfuhrdokument) anzubringen.

c) Das Eintreffen der Sendung am österreichischen Bestimmungsort ist vom Bescheidadressaten der zuständigen Bezirksverwaltungsbehörde (Amtstierärztin/Amtstierarzt) vorab bekannt zu geben.

2. **Bedingungen und Auflagen beim Grenzübertritt**

a) Jede Sendung ist beim Grenzübertritt in die EU an den Grenzkontrollstellen **Wien-Schwechat** oder **Linz** der **grenztierärztlichen Kontrolle** zu stellen.

a) Wenn der betroffene Mitgliedsstaat dem zustimmt, kann die grenztierärztliche Kontrolle an einer anderen für die Kontrolle dieser Sendungsart gemäß Artikel 59 der Verordnung (EU) 2017/625 des Europäischen Parlaments und des Rates, veröffentlicht im ABl. L 95 vom 7.4.2017, S. 1, zugelassenen und gemäß Artikel 60 dieser Verordnung veröffentlichten Grenzkontrollstelle der Europäischen Union, erfolgen.

b) Die tierärztliche Grenzkontrolle erfolgt entsprechend den rechtlichen Grundlagen der Europäischen Union, die in der Verordnung (EU) 2017/625 des Europäischen Parlaments und des Rates und allen darauf beruhenden Rechtsvorschriften festgelegt sind.

c) Die voraussichtliche Ankunft der Sendung an der veterinärbehördlichen Grenzkontrollstelle ist zumindest 1 Werktag vorher mit dem GGED (Gemeinsames Gesundheitseinfuhrdokument) anzumelden.

2. Der Bundesminister für Soziales, Gesundheit, Pflege und Konsumentenschutz behält sich vor, die Auflagen dieser Bewilligung aus veterinärpolizeilichen Gründen jederzeit zu ergänzen oder zu ändern bzw. die erteilte Bewilligung aus den gleichen Gründen entschädigungslos zurückzuziehen.

3. Die Bestätigung muss aus einem einzelnen Blatt oder einem Bogen bestehen und in einer Amtssprache jenes Mitgliedstaates, in welchem die veterinärbehördliche Grenzkontrolle stattfindet und auch in deutscher Sprache ausgestellt sein.

4. Der Bescheidadressat hat Vorsorge zu treffen, dass die Bedingungen und Auflagen dieses Bescheides den über die Sendung Verfügungsberechtigten bekannt gemacht werden.

5. Anlässlich jeder tierärztlichen Grenzkontrolle ist der Grenztierärztin/dem Grenztierarzt eine mit der Amtssignatur versehene Ausfertigung dieser Bewilligung in Form eines Ausdruckes vorzulegen.

Begründung

Gemäß § 13 der Veterinärbehördlichen Einfuhrverordnung 2022 (VEVO 2022) sind die im Spruch zitierten Tiere bewilligungspflichtig. Die veterinärbehördliche Einfuhr- bzw. Durchfuhrbewilligung ist gemäß § 14 der Veterinärbehördlichen Einfuhrverordnung 2022 durch den Bundesminister für Soziales, Gesundheit, Pflege und Konsumentenschutz auf Antrag zu erteilen, wenn mit der Einfuhr bzw. Durchfuhr der in Betracht kommenden kontrollpflichtigen Sendung die Gefahr der Einschleppung von Tierseuchen nicht verbunden ist.

Zur Verhinderung der Einschleppung von Tierseuchen war jedoch die Vorschreibung von Bedingungen und Auflagen notwendig; diese gründen sich auf die im Spruch genannten Rechtsvorschriften.

Jede Sendung unterliegt beim Grenzübergang in die EU der veterinärbehördlichen Grenzkontrolle.

Die Einfuhr der Sendung darf nur gestattet werden, wenn die Bedingungen dieses Bescheides erfüllt sind.

Dem Antrag kann vollinhaltlich entsprochen werden

Es war somit spruchgemäß zu entscheiden.

Rechtsmittelbelehrung

Gegen diesen Bescheid kann binnen vier Wochen ab Zustellung Beschwerde erhoben werden. Diese ist beim Bundesministerium für Soziales, Gesundheit, Pflege und Konsumentenschutz, Sektion III, Radetzkystraße 2, 1030 Wien, einzubringen.

Die Beschwerde hat zu enthalten:

- die Bezeichnung des angefochtenen Bescheides,
- die Bezeichnung der belangten Behörde,

- die Gründe, auf die sich die Behauptung der Rechtswidrigkeit stützt,
- das Begehren und
- die Angaben, die erforderlich sind, um zu beurteilen, ob die Beschwerde rechtzeitig eingebracht ist.

Für die Beschwerde ist eine Pauschalgebühr von € 30,- zu entrichten. Diese ist auf das Konto des Finanzamtes Österreich – Dienststelle Sonderzuständigkeit (IBAN: AT83 0100 0000 0550 4109, BIC: BUNDATWW) zu entrichten, wobei auf der Zahlungsanweisung als Verwendungszweck das jeweilige Beschwerdeverfahren (Geschäftszahl des Bescheides) anzugeben ist. Bei elektronischer Überweisung der Beschwerdegebühr mit der "Finanzamtszahlung" ist als Empfänger das Finanzamt Österreich – Dienststelle Sonderzuständigkeiten (IBAN wie zuvor) anzugeben oder auszuwählen. Weiters sind die Steuernummer 109999102, die Abgabenart "EEE - Beschwerdegebühr", das Datum des Bescheides als Zeitraum und der Betrag anzugeben.

Hinweise

1. Diese Bewilligung ersetzt nicht allenfalls erforderliche Genehmigungen gemäß anderen österreichischen bundes- und landesgesetzlichen Bestimmungen.

1. Eine Kopie dieses Bescheides wird der für den Bestimmungsort zuständigen Bezirksverwaltungsbehörde übermittelt.

2. Nähere Informationen über die österreichischen Grenzkontrollstellen finden Sie unter der Internetadresse:

   https://www.bavg.gv.at/einfuhr-import/allgemeine-informationen

Wien, 6. April 2023
Für den Bundesminister:
Mag. Georg Brandl

Beilage/n: Beilagen

REPUBLIK ÖSTERREICH BUNDESMINISTERIUM FÜR SOZIALES, GESUNDHEIT, PFLEGE UND KONSUMENTENSCHUTZ @ AMTSSIGNATUR	Unterzeichner	Bundesministerium für Soziales, Gesundheit, Pflege und Konsumentenschutz
	Datum/Zeit	2023-04-12T10:05:17+02:00
	Aussteller-Zertifikat	CN=a-sign-corporate-05,OU=a-sign-corporate-05,O=A-Trust Ges. f. Sicherheitssysteme im elektr. Datenverkehr GmbH,C=AT
	Serien-Nr.	2098721075
Hinweis	Dieses Dokument wurde amtssigniert.	
Prüfinformation	Informationen zur Prüfung des elektronischen Siegels bzw. der elektronischen Signatur finden Sie unter: http://www.signaturpruefung.gv.at<br>Informationen zur Prüfung des Ausdrucks finden Sie unter: https://www.sozialministerium.at/site/Ministerium/Willkommen_im_Ministerium/Amtssignatur/Amtssignatur	

Bundesministerium
Soziales, Gesundheit, Pflege
und Konsumentenschutz

sozialministerium.at

BMSGPK-Gesundheit - III/B/16 (Tierärztliches Berufsrecht, Tiergesundheits- und Tierschutzrecht sowie weiter rechtliche Angelegenheiten im Veterinärwesen)

Mag. Georg Brandl
Sachbearbeiter

georg.brandl@gesundheitsministerium.gv.at
+43 1 711 00-644813
Postanschrift: Stubenring 1, 1010 Wien
Radetzkystraße 2, 1030 Wien

E-Mail-Antworten sind bitte unter Anführung der Geschäftszahl an post@sozialministerium.at zu richten.

Joint FAO/IAEA Division
Insect Pest Control
zH M. Vreysen
Wagramerstrasse 5
1040 Wien

M.Vreysen@iaea.org;S.Beckham@iaea.org;B.Mayer-Karolyi@iaea.org

Geschäftszahl: 2023-0.259.084

Joint FAO/IAEA Division - Einfuhrbewilligung für Proben für Forschungs- und Diagnosezwecke aus allen Drittstaaten;
tote Puppen von Tsetsefliegen und tote Tsetsefliegen

B e s c h e i d

Der Bundesminister für Soziales, Gesundheit, Pflege und Konsumentenschutz bewilligt der Joint FAO/IAEA Division, Insect Pest Control, Wagramerstrasse 5, 1040 Wien auf Grund des Antrags vom 3. März 2023**,** gemäß §§ 4, 4a, 4b und 12a des Tierseuchengesetzes, RGBl. Nr. 177/1909, zuletzt geändert durch das Bundesgesetz BGBl. I Nr. 258/2021, in Verbindung mit §§ 13 und 14 der Veterinärbehördlichen Einfuhrverordnung 2022 (VEVO 2022), BGBl. II Nr. 480/2022, die **Einfuhr** auch partienweise von

Proben für Forschungs- und Diagnosezwecke: bestehend aus toten Puppen von Tsetsefliegen und toten Tsetsefliegen, getrocknet oder in Ethanol konserviert und in Glycerol versendet

aus **allen Drittstaaten**

nach **Österreich**
an den Bestimmungsort: **2444 Seibersdorf, Friedensstrasse 1, Insect Pest Control Laboratory der Joint FAO/IAEA Agriculture & Biotechnology Laboratories**

bis längstens **30. April 2024**

unter nachstehenden Bedingungen und Auflagen:

a) Die Einfuhr und die anschließende Lagerung am Bestimmungsort, müssen in undurchlässigen Behältern erfolgen. Die Verpackung muss mindestens dreischichtig sein, zwei Schichten müssen undurchlässig, druckfest und stoßfest sein. Die Behälter müssen von saugfähigem Material umgeben und versiegelbar sein.
b) Jeder Behälter muss entsprechend den internationalen Vorschriften gekennzeichnet sein (z.B. UN 3373, UN 2814 oder UN 2900).
c) Die Behälter oder ihre äußere Verpackung müssen die deutlich erkennbare Aufschrift „Nur zur Verwendung im Labor“ oder “For laboratory use only” tragen.
d) Jede Sendung muss von der Zollstelle in einem geeigneten Verkehrsmittel unmittelbar zum Bestimmungsort gebracht werden.
e) Jeder Verwender hat die Punkte f) und g) zu beachten dies gilt auch, wenn die eingeführten Proben nicht am Bestimmungsort verwendet werden.
f) Alle erforderlichen Arbeiten sind so durchzuführen, dass eine Gefährdung des österreichischen Tierbestandes und der Menschen mit Sicherheit auszuschließen ist.
g) Die Verpackungen sowie nicht benötigtes Forschungsmaterial sind jeweils seuchensicher über ein zugelassenes Entsorgungssystem für klinischen oder Labor-Abfall bzw. gemäß der VO (EG) Nr. 1069/2009 als Material der Kategorie 1 durch Verbrennen zu entsorgen.

1. **Kontrolle sowie Bedingungen und Auflagen beim Grenzübertritt**

a) Die Kontrolle erfolgt in Österreich **durch die Zollbehörden**; eine Verbringung in die Europäische Union (EU) ist daher über jede österreichische Zollstelle möglich.
a) Es ist keine Kontrolle durch die Grenztierärztinnen/die Grenztierärzte erforderlich.
b) Erfolgt die Verbringung in die EU nicht über eine österreichische Zollstelle, ist die Kontrolle an einer zugelassenen Grenzkontrollstelle unter Vorlage dieser Bewilligung durchführen zu lassen, an der eine IMSOC/TRACES Meldung abgesetzt werden kann.

2. Der Bundesminister für Soziales, Gesundheit, Pflege und Konsumentenschutz behält sich vor, die Auflagen dieser Bewilligung aus veterinärpolizeilichen Gründen jederzeit zu ergänzen oder zu ändern bzw. die erteilte Bewilligung aus den gleichen Gründen entschädigungslos zurückzuziehen.

3. Der Bescheidadressat hat Vorsorge zu treffen, dass die Bedingungen und Auflagen dieses Bescheides den jeweils über die Sendung Verfügungsberechtigten bekannt gemacht werden.

4. Eine mit der Amtssignatur versehene Ausfertigung ist den Zollorganen bei der Kontrolle jeder Sendung in Form eines Ausdruckes vorzulegen. Bei der Überführung der Waren in ein Zollverfahren, einschließlich der Verbringung in Zolllager oder Freizonen und dem Versandverfahren, ist die vorliegende Bewilligung im Feld 44 der Zollanmeldung durch den Code „7299“ samt deren Geschäftszahl anzugeben.

B e g r ü n d u n g

Gemäß § 13 der Veterinärbehördlichen Einfuhrverordnung sind die im Spruch zitierten Waren bewilligungspflichtig. Die veterinärbehördliche Einfuhr- bzw. Durchfuhrbewilligung ist gemäß § 14 durch den Bundesminister für Soziales, Gesundheit, Pflege und Konsumentenschutz auf Antrag zu erteilen, wenn mit der Einfuhr bzw. Durchfuhr der in Betracht kommenden kontrollpflichtigen Sendung die Gefahr der Einschleppung von Tierseuchen nicht verbunden ist.

Zur Verhinderung der Einschleppung von Tierseuchen war jedoch die Vorschreibung von Bedingungen und Auflagen notwendig; diese gründen sich auf die im Spruch genannten Rechtsvorschriften.

Jede Sendung unterliegt beim Grenzübertritt in die EU der Grenzkontrolle durch die zuständige Behörde; in Österreich erfolgt die Kontrolle bei den im Spruch genannten Waren durch die Zollbehörde.

Die Einfuhr der Sendung darf nur gestattet werden, wenn die Bedingungen dieses Bescheides erfüllt sind.

Dem Antrag des Bescheidadressaten kann vollinhaltlich entsprochen werden.

Es war somit spruchgemäß zu entscheiden.

R e c h t s m i t t e l b e l e h r u n g

Gegen diesen Bescheid kann binnen vier Wochen ab Zustellung Beschwerde erhoben werden. Diese ist beim Bundesministerium für Soziales, Gesundheit, Pflege und Konsumentenschutz, Sektion III, Radetzkystraße 2, 1030 Wien, einzubringen.

Die Beschwerde hat zu enthalten:

- die Bezeichnung des angefochtenen Bescheides,
- die Bezeichnung der belangten Behörde,
- die Gründe, auf die sich die Behauptung der Rechtswidrigkeit stützt,
- das Begehren und
- die Angaben, die erforderlich sind, um zu beurteilen, ob die Beschwerde rechtzeitig eingebracht ist.

Für die Beschwerde ist eine Pauschalgebühr von € 30,- zu entrichten. Diese ist auf das Konto des Finanzamtes Österreich – Dienststelle Sonderzuständigkeit (IBAN: AT83 0100 0000 0550 4109, BIC: BUNDATWW) zu entrichten, wobei auf der Zahlungsanweisung als Verwendungszweck das jeweilige Beschwerdeverfahren (Geschäftszahl des Bescheides) anzugeben ist. Bei elektronischer Überweisung der Beschwerdegebühr mit der "Finanzamtszahlung" ist als Empfänger das Finanzamt Österreich – Dienststelle Sonderzuständigkeiten (IBAN wie zuvor) anzugeben oder auszuwählen. Weiters sind die Steuernummer 109999102, die Abgabenart "EEE - Beschwerdegebühr", das Datum des Bescheides als Zeitraum und der Betrag anzugeben.

H i n w e i s e

1. Diese Bewilligung ersetzt nicht allenfalls erforderliche Genehmigungen gemäß anderen österreichischen bundes- und landesgesetzlichen Bestimmungen.

2. Eine Kopie dieses Bescheides wird der für den Bestimmungsort zuständigen Bezirksverwaltungsbehörde übermittelt.

Wien, 6. April 2023
Für den Bundesminister:
Mag. Georg Brandl

Beilage/n: Beilagen

REPUBLIK ÖSTERREICH BUNDESMINISTERIUM FÜR SOZIALES, GESUNDHEIT, PFLEGE UND KONSUMENTENSCHUTZ AMTSSIGNATUR	Unterzeichner	Bundesministerium für Soziales, Gesundheit, Pflege und Konsumentenschutz
	Datum/Zeit	2023-04-12T10:29:54+02:00
	Aussteller-Zertifikat	CN=a-sign-corporate-05,OU=a-sign-corporate-05,O=A-Trust Ges. f. Sicherheitssysteme im elektr. Datenverkehr GmbH,C=AT
	Serien-Nr.	2098721075
Hinweis	Dieses Dokument wurde amtssigniert.	
Prüfinformation	Informationen zur Prüfung des elektronischen Siegels bzw. der elektronischen Signatur finden Sie unter: http://www.signaturpruefung.gv.at<br>Informationen zur Prüfung des Ausdrucks finden Sie unter: https://www.sozialministerium.at/site/Ministerium/Willkommen_im_Ministerium/Amtssignatur/Amtssignatur	
